# ChronoSphere: a graph-based EMF model repository for IT landscape models

**DOI:** 10.1007/s10270-019-00725-0

**Published:** 2019-03-08

**Authors:** Martin Haeusler, Thomas Trojer, Johannes Kessler, Matthias Farwick, Emmanuel Nowakowski, Ruth Breu

**Affiliations:** 1 University of Innsbruck, 6020 Innsbruck, Austria; 2 Txture GmbH, 6020 Innsbruck, Austria

**Keywords:** Model-driven engineering, Model repositories, Versioning, Graph database, IT landscape

## Abstract

IT Landscape models are representing the real-world IT infrastructure of a company. They include hardware assets such as physical servers and storage media, as well as virtual components like clusters, virtual machines and applications. These models are a critical source of information in numerous tasks, including planning, error detection and impact analysis. The responsible stakeholders often struggle to keep such a large and densely connected model up-to-date due to its inherent size and complexity, as well as due to the lack of proper tool support. Even though modeling techniques are very suitable for this domain, existing tools do not offer the required features, scalability or flexibility. In order to solve these challenges and meet the requirements that arise from this application domain, we combine domain-driven modeling concepts with scalable graph-based repository technology and a custom language for model-level queries. We analyze in detail how we synthesized these requirements from the application domain and how they relate to the features of our repository. We discuss the architecture of our solution which comprises the entire data management stack, including transactions, queries, versioned persistence and metamodel evolution. Finally, we evaluate our approach in a case study where our open-source repository implementation is employed in a production environment in an industrial context, as well as in a comparative benchmark with an existing state-of-the-art solution.

## Introduction

Model-driven engineering (MDE) is a discipline that aims at improving the processes, workflows and products of existing engineering areas by applying models as an abstraction layer. The primary field of application for MDE has traditionally always been software engineering [64]. However, the key innovations of MDE are not domain specific. The general concept of using a metamodel to define a structure and then instantiating it to create actual objects applies to a wide range of problems. When comparing different use cases it becomes evident that modeling concepts tend to be employed in areas that exhibit high complexity and heterogeneity in their domain structures, such as cloud orchestration [22], self-adaptive software [5], automotive systems [24] or Enterprise Architecture Management (EAM) [43]. However, there are still many potential application areas for model-driven approaches that have barely been investigated so far. EAM focuses exclusively on the strategic aspects of IT management. Standard metamodels (such as ArchiMate [43]) have been developed for this domain, yet these metamodels focus primarily on high-level business services and capabilities. The actual assets (or *Configuration Items (CIs)* [7]) on the operative level are captured in a coarse-grained way that does not allow for deeper analysis, or are excluded entirely.

Configuration items typically comprise physical servers, applications, databases and network infrastructure. We refer to the collection of all assets in a company as the *IT Landscape* (also known as *resource landscape* [36]). The IT Landscapes of major, globally operating companies, can grow to considerable dimensions. Due to agility requirements, they are increasingly subject to frequent evolution and technology shifts. Recent examples include the extensive usage of virtualization platforms in data centers, the advent of cloud computing and the emergence of As-A-Service solutions. Furthermore, even though commonalities do exist, every company has its own architecture and vision behind its landscape. The terminology also varies, as there is no generally accepted definition across all stakeholders for common terms like *Service* or *Application*. Responsible persons and teams often struggle in their continuous efforts to properly document these landscapes due to their inherent size and complexity. The absence of a reliable and up-to-date documentation can result in slow error detection, loss of traceability of changes and misguided planning processes due to poor information situations. Ultimately, these issues can lead to problems which cause very high costs for the companies if they remain unaddressed [30, 51].

The need for tool support in the area of IT Landscape documentation is evident, and model engineering is well-suited to provide the required concepts. However, the existing MDE tool infrastructure is insufficient when it comes to satisfying the requirements of this domain. Existing solutions either do not scale with the number of elements in a real-world IT Landscape documentation, do not offer the necessary analysis capabilities, or lack the flexibility needed in long-term projects. Several state-of-the-art model repositories employ relational databases, even though the object-relational gap is well-known to cause additional complexity and performance overhead. Furthermore, the required commitment to a fixed schema across all entries impedes the ability to perform metamodel evolution processes without altering past revisions. In recent years, the NoSQL family of databases has expanded, and graph databases in particular are an excellent fit for storing model data [1, 4]. The central research question we focus on in this paper is how to combine domain-driven modeling concepts and technologies with the innovations from the graph database community in order to build a model repository which is suitable for IT Landscape documentation.

In this paper, we present a solution for storing, versioning and querying IT Landscape models called *ChronoSphere*. ChronoSphere is a novel open-source EMF model repository that addresses the needs of this domain, in particular scalable versioning, querying and persistence. It utilizes innovative database technology and is based on a modular architecture which allows individual elements to be used as standalone components outside the repository context. Even though ChronoSphere has been designed for the IT Landscape use case, the core implementation is domain independent and may also serve other use cases (see Sect. 9.4). In our inter-disciplinary efforts to realize this repository, we also contributed to the state-of-the-art in the database community, in particular in the area of versioned data storage and graph versioning. We evaluate our approach in an industrial case study in collaboration with *Txture GmbH*.^1^ This company employs our ChronoSphere implementation as the primary storage back-end in their commercial IT Landscape modeling tool.

The remainder of this paper is structured as follows. In Sect. 2, we first describe the IT Landscape use case in more detail. We then extract the specific requirements for our solution from this environment and discuss how they were derived from the industrial context. Section 3 provides a high-level overview of our approach. In Sects. 4 through 6, we discuss the details of our solution. In Sect. 7, we present the application of our repository in an industrial context. Section 8 evaluates the performance of ChronoSphere in comparison with other model repository solutions, which is followed by a feature-based comparison of related work in several different areas in Sect. 9. We conclude the paper with an outlook to future work in Sect. 10 and a summary in Sect. 11. Sections 4 through 6 consist of a revised, updated and extended version of the content presented in our previous work, mainly [25, 27, 28]. The remaining sections (most notably 2, 7 and 8) have never been published before.

## Use case and requirement analysis

The overarching goal in IT Landscape documentation is to produce and maintain a model which reflects the current IT assets of a company and their relationships with each other. As these assets change over time, keeping this model up-to-date is a continuous task, rather than a one-time effort.

**Fig. 1:**
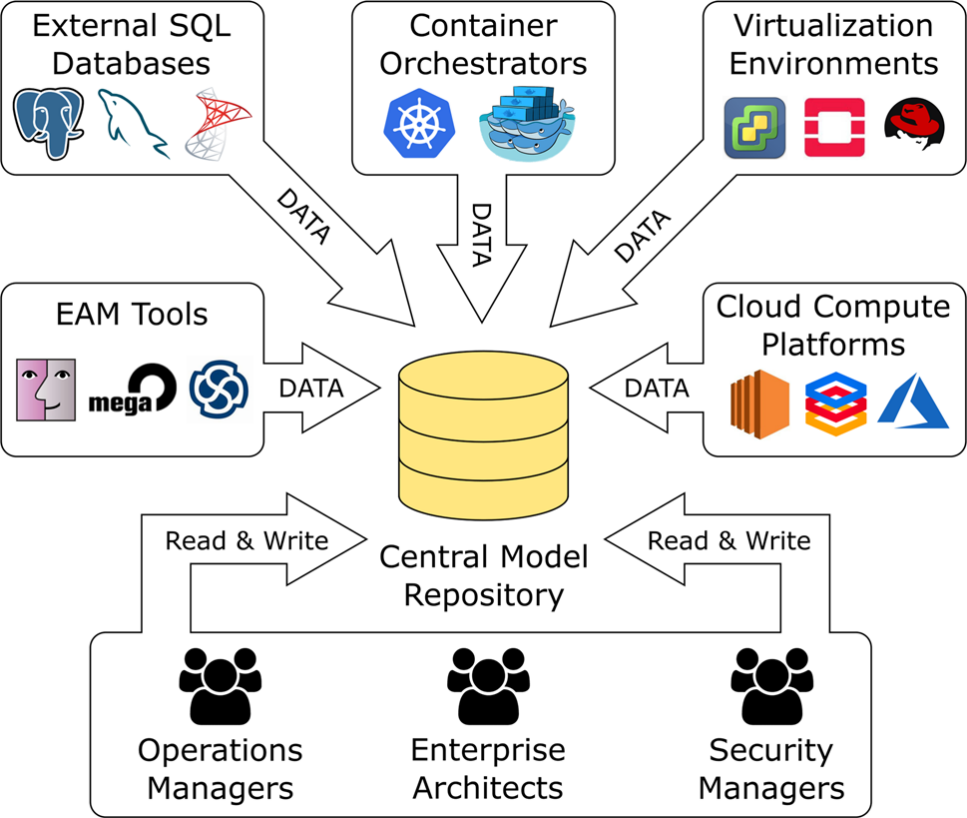
The IT landscape environment

**Table 1:** Common data sources for IT landscape documentation

Data source	Examples	Web URL
SQL databases	MySQL	www.mysql.com
	PostGreSQL	www.postgresql.org
	Microsoft SQL Server	www.microsoft.com/en-us/sql-server
Virtualization platforms	VMware VCenter	www.vmware.com/products/vcenter-server
	OpenStack	www.openstack.org
	Red Hat Enterprise Virtualization	www.redhat.com/en/technologies/virtualization
Enterprise architecture management tools	IteraPlan	www.iteraplan.de/en
	Mega	www.mega.com/en/product/enterprise-architecture
	Enterprise Architect	www.sparxsystems.eu
Cloud Computation Platforms	Amazon EC2	https://aws.amazon.com/ec2
	Microsoft Azure	https://azure.microsoft.com/en-us
	Google Cloud Compute Engine	https://cloud.google.com/compute
Container orchestration mechanisms	Google Kubernetes	https://kubernetes.io
	Docker Swarm	https://docs.docker.com/engine/swarm

From a repository perspective, the use case of IT Landscape documentation is unique because it is both a database scenario (involving large datasets) as well as a design scenario where multiple users manually edit the model in a concurrent fashion (see Fig. 1). The amount and quality of information which is available in external data sources depends on the degree of automation and standardization in the company. For companies with a lower degree of automation, users will want to edit the model manually to keep it up-to-date. In companies that have a sophisticated automation chain in place, the majority of data can be imported into the repository without manual intervention. Typical data sources involved in such a scenario are listed in Table 1.

After gathering and consolidating the required information in a central repository, typical use cases are centered around analysis and reporting. A user usually starts a session with a query that finds all assets that match a list of criteria, such as “Search for all Virtual Machines which run a Linux Operating System” or “Search for the Cluster named ‘Production 2’ located in Vienna”. Finding an asset based on its name is a particularly common starting query.

From the result of this initial global query, the user will often want to analyze this particular asset or group of assets. Common use cases involve impact analysis and root cause analysis. The central question in impact analysis is “What would be the impact to my applications if a given Physical Server fails” and can be answered by a transitive dependency analysis starting from the Physical Server and resolving the path to the transitively connected applications (crossing the virtualization, clustering and load balancing layers in between). Root cause analysis is the inverse question: given an Application, the task is to find all Physical Servers on which the application transitively depends. This insight allows to reduce the search space in case of an incident (ranging from performance problems to total application outage).

Finally, analyzing the history of a single element or the entire model as a whole are important use cases in IT Landscape management. For example, users are interested in the number of applications employed in their company over time. Version control becomes essential in such scenarios, because it allows to formulate queries over time after the actual insertion of the data has happened (whereas for a statistic on a non-versioned store the query would have to be known in advance to track the relevant data at insertion time). Per-element history traces are also important, as they allow to identify who performed a certain change, which properties of the asset have been modified, and when the modification has occurred.

**Fig. 2:**
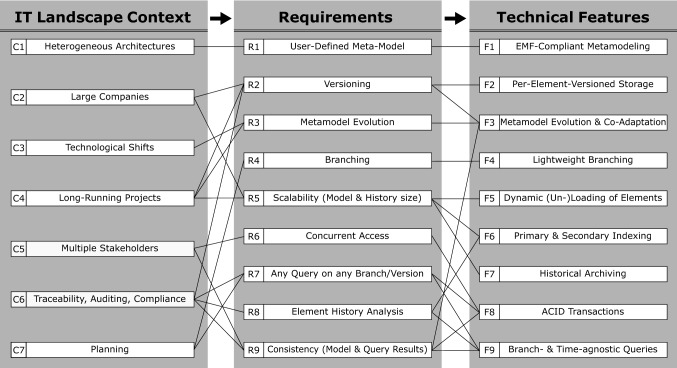
Traceability matrix between context, requirements and features

In the remainder of this section, we focus on the most important influences from the industrial context, how we derived requirements for our repository from them, and how these requirements are met by technical features. Figure 2 provides an overview of this process.

### Deriving requirements from the context

IT architectures and their terminology (e.g., the exact definition of general terms like *Service* or *Application*) vary by company. Therefore, the *structure* of the resulting IT Landscape models also differs. One solution to these *Heterogeneous Architectures* [C1] is to unify them under a common, fixed metamodel (e.g., ArchiMate [43]). However, this can lead to poor acceptance in practice due to its rigidity and the additional complexity introduced by its generality. From our past surveys and case studies [17–20, 73], we inferred the requirement that the metamodel should be configurable by the user [R1]. The companies which utilize IT Landscape models the most are usually large companies [C2], or companies with a strong focus on IT (such as data centers). This entails that the corresponding models will grow to considerable sizes, and a repository must offer the necessary scalability [R5].

Documenting the IT Landscape of a company is a continuous effort. In the industrial context, we are therefore faced with long-running endeavors [C4] that span several years. In situations where responsible persons change and team members leave while new ones join, the ability to comprehend and reflect upon past decisions becomes crucial. Versioning the model content [R2] meets these demands, and also enables use cases that involve auditing, as well as use cases where versioning is required for legal compliance [C6]. The underlying requirement for these use cases is to not only store the version history, but also to analyze history traces [R8]. During a long-term documentation project, the metamodel sometimes also needs to be adapted [R3], for example due to technological shifts [C3] that introduce new types of assets. Examples for technological shifts include the introduction of virtualization technologies in data centers, and the advent of cloud computing. Another direct consequence of long-running projects is that the change history of individual model elements can grow to large sizes [R5] which must be considered in the technical realization of a repository.

In industrial contexts, several different stakeholders collaborate in documenting the IT Landscape. Depending on the scope of the project, stakeholders can involve a wide variety of roles, ranging from IT operations experts to database managers and enterprise architects [C5]. This entails that the repository must support concurrent access [R6] for multiple users. Another requirement that follows directly from concurrent access is that the structural consistency of the model contents must be ensured by the repository [R6, R9] (e.g., conformance to the metamodel and referential integrity). From the analysis perspective, concurrent access is also a threat to the consistency and reproducibility of query results, which is required for reliable model analysis [R9]. Apart from analyzing the current and past states of the model, IT Landscape models are also used to plan for future transformations [C7]. The general workflow involves the creation of “to-be” scenarios based on the current state which are then compared against each other in order to select the best candidate. In order to cope with such use cases, the repository must support branching [R4]. Branches allow the plans to be based on the actual model and to change it independently without affecting the as-is state. Since the comparison of two model states is an important part of planning (as well as a number of other use cases), the repository needs to be able to evaluate an analysis query on any model version and on any branch [R7] without altering the query.

### Deriving features from requirements

From the set of requirements, we inferred the technical features which our model repository has to support. As we want our users to be able to provide their own metamodels, we employ the standard Eclipse Modeling Framework (EMF [70]) as our modeling language of choice [F1]. The fact that the data managed by our repository consists of a large and densely connected model which has to be put under version control lead to the decision to employ a per-element versioning strategy [F2], as a coarse-grained whole-model versioning strategy would cause performance issues for such models.

Supporting a user-defined metamodel, element versioning and metamodel evolution at the same time is a challenging task. The combination of these requirements entails that our repository must support metamodel versioning, metamodel evolution and instance co-adaptation [F3]. From a technical perspective, it is also inadvisable to create a full copy of a model version each time a branch is created due to the potential size of the model. We therefore require a branching mechanism that is *lightweight* [F4] in that it reuses the data from the origin branch rather than copying it when a new branch is created. Since IT Landscape models can grow to large sizes and will potentially not fit into the main memory of the machine which runs our repository, we require a mechanism for dynamic on-demand loading and unloading of model elements [F5].

A technical feature which is crucial for efficient querying of the entire model is indexing [F6]. The *primary index* allows to locate a model element by its unique ID without linear iteration over all elements. *Secondary indices* can be defined by the user for a given metamodel and can be used to efficiently find all elements in the model where a property is set to a specified value (e.g., finding all servers where the name contains “Production”). In addition, indexing has to consider the versioned nature of our repository, as we want our indices to be usable for *all* queries, regardless of the chosen version or branch. In other words, even a query on a model version that is one year old should be able to utilize our indices. In order for queries to be executable on any branch and timestamp, we require a framework that allows for the creation of queries that are agnostic to the chosen branch and version [F9].

All queries and modifications in our repository are subject to concurrent access. We meet this requirement by providing full ACID [38] end-to-end transaction support in our repository [F8]. Finally, in order to support long histories, we implement a feature called Temporal Rollover which enables the archiving of historical entries [F7]. This feature allows for indefinite growth of element histories and will be explained in detail in later sections.

## Solution overview

The overarching goal of our efforts is to provide a model repository that fulfills the requirements in Sect. 2. Our initial prototypes were based on standard technology, such as object-relational mappers and SQL databases. However, we soon realized that table-based representations were not an ideal fit for the structure of model data. The main issues we faced with these solutions were related to scalability and performance [R5]. The fact that most SQL databases require a fixed schema also proved to be very limiting when taking the requirement for metamodel evolution [R3] into consideration.

During our search for alternatives, we were inspired by approaches such as MORSA [54] and Neo4EMF [4]. We investigated various NoSQL storage solutions and eventually settled for graph databases. Graph databases do not require a fixed schema, offer fast execution of navigational queries and the bijective mapping between model data and graph data is both simpler and faster than object-relational mappings. However, existing graph databases on the market did not offer built-in versioning capabilities [R2]. Using a general-purpose graph database (e.g., Neo4j^2^ or Titan^3^) and managing the versioning process entirely on the application side has already been proven by various authors to be possible [8, 66, 67, 71]. However, such approaches greatly increase the complexity of the resulting graph structure as well as the complexity of the queries that operate on it. This reduces the maintainability, performance and scalability [R5] of such systems.

**Fig. 3:**
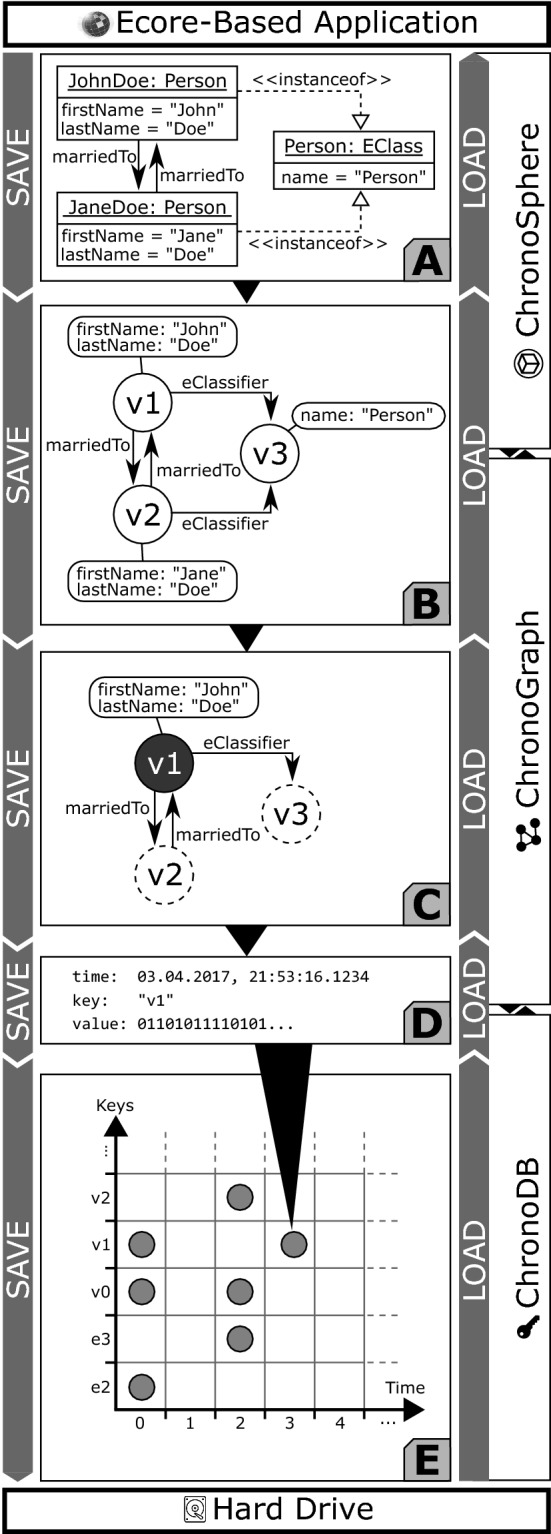
ChronoSphere data management stack

After discovering this gap in both research and industrial solutions, we created a versioned graph database called *ChronoGraph* [27], which is the first graph database with versioning support that is compliant to the *Apache TinkerPop* standard. ChronoGraph is listed^4^ as an official TinkerPop implementation and available as an open-source project on GitHub.^5^ Since graph structures need to be transformed into a format that is compatible with the sequential nature of hard drives, we also required a versioned storage solution. Our explorative experiments with different back-ends of Titan DB demonstrated that key-value stores are a very suitable fit for storing graph data. We created *ChronoDB* [25], a versioned key-value store, to act as the storage backend for ChronoGraph. The full source code of this project is also available on GitHub.^6^ The resulting repository solution therefore consists of three layers, allowing for a coherent architecture and a clean separation of concerns.

Figure 3 shows the data management concepts of ChronoSphere. At the very top, in Fig. 3 Part *A*, we are working with EObjects and their corresponding EClasses, EPackages and other Ecore elements. It is important that the model and the metamodel are stored *together*. This will become a critical factor when dealing with metamodel evolution. This combined model needs to be persisted and versioned [R1–R4]. ChronoSphere maps it to a property graph representation [58] for this purpose. This representation is conceptually very close to the model form. Our model-to-graph mapping is inspired by Neo4EMF [4]. We will discuss related work in this area in more detail in Sect. 9.

The property graph management in Fig. 3 Part *B* is provided by ChronoGraph. In order to achieve a serial form for the model data that can be persisted to disk, ChronoGraph disassembles the property graph into individual *Star Graphs*, one for each vertex (i.e., node). A star graph is a sub-graph that is centered around one particular vertex. Figure 3 Part *C* shows the star graph of vertex *v*1. Creating star graphs for each vertex is a special kind of *graph partitioning*. When linking the star graphs again by replacing IDs by vertices, the original graph can be reconstructed from this partitioning. This reconstruction can occur fully or only partially, which makes this solution very suitable for lazy loading techniques [R5].

In the next step, we transform the star graph of each vertex into a binary string using the *Kryo*^7^ serializer, and pass the result to the underlying ChronoDB, our versioned Key-Value-Store. When the transaction is committed [R6], the commit timestamp is assigned to each pair of modified keys and corresponding binary values, creating time-key-value triples as shown in Fig. 3 Part *D*. ChronoDB then stores these triples in a *Temporal Data Matrix* (Fig. 3 Part *E*) which is implemented as a B$^{+}$-Tree [61]. Each row in this matrix represents the full history of a single element, each column represents a model revision, and each cell represents the data of one particular element for a given ID at a given timestamp. We will define and discuss this matrix structure in more detail in the following section.

## Solution part I: ChronoDB

ChronoDB [25] is a versioned key-value store and the bottom layer in our architecture. Its main responsibilities are persistence, versioning, branching and indexing. As all other components in our architecture rely on this store, we formalized its data structures and operations during the design phase.

### Formal foundation

Salzberg and Tsotras identified three key query types which have to be supported by a data store in order to provide the full temporal feature set [62]. For versioning purposes, this set can be reused by restricting the features to timestamps instead of time ranges. This gives rise to the following three types of possible queries:

- *Pure-Timeslice Query* Given a point in time (e.g., date and time), find all keys that existed at that time.
- *Range-Timeslice Query* Given a set of keys and a point in time, find the value for each key which was valid at that time.
- *Pure-Key Query* Given a set of keys, for each key find the values that comprise its history.

We use these three core query types as the functional requirements for our formalization approach. For practical reasons, we furthermore require that inserted entries never have to be modified again. In this way, we can achieve a true *append-only* store. In order to maintain the traceability of changes over time (e.g., for auditing purposes [R8]), we also require that the history of a key must never be altered, only appended.

The key concept behind our formalism is based on the observation that temporal information always adds an additional dimension to a dataset. A key-value format has only one dimension, which is the key. By adding temporal information, the two resulting dimensions are the key, and the time at which the value was inserted. Therefore, a matrix is a very natural fit for formalizing the versioning problem, offering the additional advantage of being easy to visualize. The remainder of this section consists of definitions which provide the formal semantics of our solution, interleaved with figures and (less formal) textual explanations.

#### Definition 1

Temporal Data Matrix

Let *T* be the set of all timestamps with $T \subseteq \mathbb {N}$. Let $\mathcal {S}$ denote the set of all non-empty strings and *K* be the set of all keys with $K \subseteq \mathcal {S}$. Let $\mathbb {B}$ define the set of all binary strings with $\mathbb {B} \subseteq \{0,1\}^+ \cup \{null, \epsilon \}$. Let $\epsilon \in \mathbb {B}$ be the empty binary string with $\epsilon \ne null$. We define the *Temporal Data Matrix*$\mathcal {D} \in \mathbb {B}^{\infty \times \infty }$ as:

$$\begin{aligned} \mathcal {D}: T \times K \rightarrow \mathbb {B} \end{aligned}$$

We define the initial value of a given Temporal Data Matrix *D* as:

$$\begin{aligned} D_{t,k} := \epsilon \qquad \forall t \in T, \forall k \in K \end{aligned}$$

In Definition 1, we define a Temporal Data Matrix, which is a key-value mapping enhanced with temporal information [R2, R3]. Note that the number of rows and columns in this matrix is infinite. In order to retrieve a value from this matrix, a key string and a timestamp are required. We refer to such a pair as a *Temporal Key*. The matrix can contain an array of binary values in every cell, which can be interpreted as the serialized representation of an arbitrary object. The formalism is therefore not restricted to any particular value type. The dedicated *null* value (which is different from all other bit-strings and also different from the $\epsilon$ values used to initialize the matrix) will be used as a marker that indicates the deletion of an element later in Definition 3.

In order to guarantee the traceability of changes [R8], entries in the past must not be modified, and new entries may only be appended to the end of the history, not inserted at an arbitrary position. We use the notion of a dedicated *now* timestamp for this purpose.

#### Definition 2

Now Operation

Let *D* be a Temporal Data Matrix. We define the function $now: \mathbb {B}^{\infty \times \infty } \rightarrow T$ as:

$$\begin{aligned} now(D) = max(\{t | k \in K, D_{t,k} \ne \epsilon \} \cup \{0\}) \end{aligned}$$

Definition 2 introduces the concept of the *now* timestamp, which is the largest (i.e., latest) timestamp at which data has been inserted into the store so far, initialized at zero for empty matrices. This particular timestamp will serve as a safeguard against temporal inconsistencies in several operations. We continue by defining the temporal counterparts of the *put* and *get* operations of a key-value store.

#### Definition 3

Temporal Write Operation

Let *D* be a Temporal Data Matrix. We define the function $put: \mathbb {B}^{\infty \times \infty } \times T \times K \times \mathbb {B} \rightarrow \mathbb {B}^{\infty \times \infty }$ as:

$$\begin{aligned} put(D,t,k,v) = D' \end{aligned}$$

with $$v \ne \epsilon $$, $$t > now(D)$$ and

$$\begin{aligned} D'_{i,j} := {\left\{ \begin{array}{ll} v &{} \hbox {if } t = i \wedge k = j\\ D_{i,j} &{} \hbox {otherwise}\\ \end{array}\right. } \end{aligned}$$

The write operation *put* replaces a single entry in a Temporal Data Matrix by specifying the exact coordinates and a new value for that entry. All other entries remain the same as before. Please note that, while *v* must not be $\epsilon$ in the context of a *put* operation (i.e., a cell cannot be “cleared”), *v* can be *null* to indicate a deletion of the key *k* from the matrix. Also, we require that an entry must not be overwritten. This is given implicitly by the fact that each *put* advances the result of *now*(*D*), and further insertions are only allowed after that timestamp. Furthermore, write operations are not permitted to modify the past in order to preserve consistency and traceability, which is also asserted by the condition on the *now* timestamp. This operation is limited in that it allows to modify only one key at a time. In the implementation, we generalize it to allow simultaneous insertions in several keys via transactions.

#### Definition 4

Temporal Read Operation

Let *D* be a Temporal Data Matrix. We define the function $get: \mathbb {B}^{\infty \times \infty } \times T \times K \rightarrow \mathbb {B}$ as:

$$\begin{aligned} get(D,t,k) := {\left\{ \begin{array}{ll} D_{u,k} &{} \text{ if } u \ge 0 \wedge D_{u,k} \ne null\\ \epsilon &{} \text{ otherwise } \end{array}\right. } \end{aligned}$$

with $$t \le now(D)$$ and

$$\begin{aligned} u := max(\{x | x \in T, x \le t, D_{x,k} \ne \epsilon \} \cup \{-1\}) \end{aligned}$$

The function *get* first attempts to return the value at the coordinates specified by the key and timestamp ($u = t$). If that position is empty, we scan for the entry in the same row with the highest timestamp and a non-empty value, considering only entries with lower timestamps than the request timestamp. In the formula, we have to add $-~1$ to the set from which *u* is chosen to cover the case where there is no other entry in the row. If there is no such entry (i.e., $u = -~1$) or the entry is *null*, we return the empty binary string, otherwise we return the entry with the largest encountered timestamp.

**Fig. 4:**
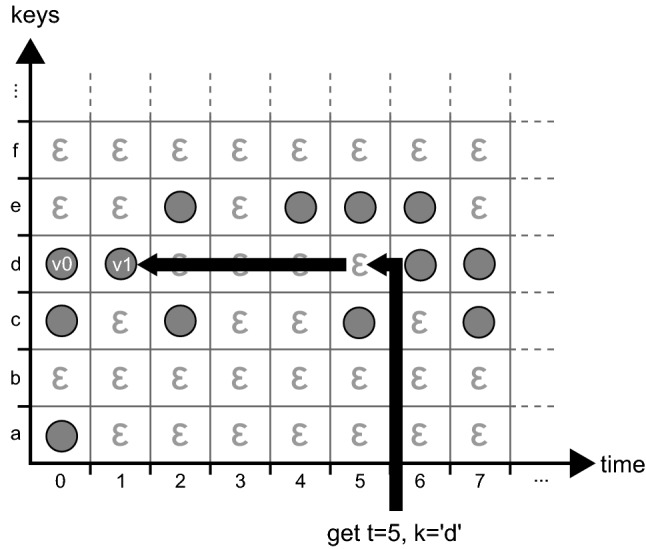
A *get* operation on a Temporal Data Matrix [25]

This process is visualized in Fig. 4. In this figure, each row corresponds to a key, and each column to a timestamp. The depicted *get* operation is working on timestamp 5 and key ‘d’. As $D_{5,d}$ is empty, we attempt to find the largest timestamp smaller than 5 where the value for the key is not empty, i.e., we move left until we find a non-empty cell. We find the result in $D_{1, d}$ and return *v*1. This is an important part of the versioning concept: a value for a given key is assumed to remain unchanged until a new value is assigned to it at a later timestamp. This allows any implementation to conserve memory on disk, as writes only occur if the value for a key has changed (i.e., no data duplication is required between identical revisions). Also note that we do not need to update existing entries when new key-value pairs are being inserted, which allows for pure *append-only* storage. In Fig. 4, the value *v*1 is valid for the key ‘d’ for all timestamps between 1 and 5 (inclusive). For timestamp 0, the key ‘d’ has value *v*0. Following this line of argumentation, we can generalize and state that a *row* in the matrix, identified by a key $k \in K$, contains the *history* of *k*. This is formalized in Definition 5. A column, identified by a timestamp $t \in T$, contains the state of all keys at that timestamp, with the additional consideration that value duplicates are not stored as they can be looked up in earlier timestamps. This is formalized in Definition 6.

#### Definition 5

History Operation

Let *D* be a Temporal Data Matrix, and *t* be a timestamp with $t \in T, t \le now(D)$. We define the function $history: \mathbb {B}^{\infty \times \infty } \times T \times K \rightarrow 2^{T}$ as:

$$\begin{aligned} history(D,t,k) := \{x | x \in T, x \le t, D_{x,k}\ne \epsilon \} \end{aligned}$$

In Definition 5, we define the *history* of a key *k* up to a given timestamp *t* in a Temporal Data Matrix *D* as the set of timestamps less than or equal to *t* that have a non-empty entry for key *k* in *D*. Note that the resulting set will also include deletions, as *null* is a legal value for $D_{x,k}$ in the formula. The result is the set of timestamps where the value for the given key changed. Consequently, performing a *get* operation for these timestamps with the same key will yield different results, producing the full history of the temporal key.

#### Definition 6

Keyset Operation

Let *D* be a Temporal Data Matrix, and *t* be a timestamp with $t \in T, t \le now(D)$. We define the function $keyset: \mathbb {B}^{\infty \times \infty } \times T \rightarrow 2^{K}$ as:

$$\begin{aligned} keyset(D,t) := \{x | x \in K, get(D,t,x)\ne \epsilon \} \end{aligned}$$

As shown in Definition 6, the keyset in a Temporal Data Matrix changes over time. We can retrieve the keyset at any desired time by providing the appropriate timestamp *t*. Note that this works for any timestamp in the past, in particular we do not require that a write operation has taken place precisely at *t* in order for the corresponding key(s) to be contained in the keyset. In other words, the precise column of *t* may consist only of $\epsilon$ entries, but the key set operation will also consider earlier entries which are still valid at *t*. The version operation introduced in Definition 7 operates in a very similar way, but returns tuples containing keys and values, rather than just keys.

#### Definition 7

Version Operation

Let *D* be a Temporal Data Matrix, and *t* be a timestamp with $t \in T, t \le now(D)$. We define the function $version: \mathbb {B}^{\infty \times \infty } \times T \rightarrow 2^{K \times \mathbb {B}}$

$$\begin{aligned} version(D,t) := \{\langle k,v\rangle | k \in keyset(D,t), v = get(D,t,k)\} \end{aligned}$$

**Fig. 5:**
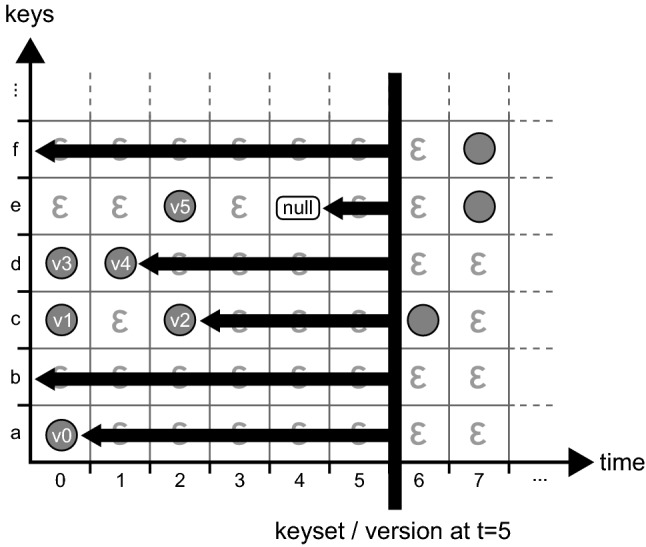
Performing a *keyset* or *version* operation on a Temporal Data Matrix [25]

Figure 5 illustrates the key set and version operations by example. In this scenario, the key set (or version) is requested at timestamp $t = 5$. We scan each row for the latest non-$\epsilon$ entry and add the corresponding key of the row to the key set, provided that a non-$\epsilon$ right-most entry exists (i.e., the row is not empty) and is not *null* (the value was not removed). In this example, *keyset*(*D*, 5) would return $\{a,c,d\}$, assuming that all non-depicted rows are empty. *b* and *f* are not in the key set, because their rows are empty (up to and including timestamp 5), and *e* is not in the set because its value was removed at timestamp 4. If we would request the key set at timestamp 3 instead, *e* would be in the key set. The operation *version*(*D*, 5) returns $\{ \langle a,v0\rangle , \langle c, v2\rangle , \langle d, v4\rangle \}$ in the example depicted in Fig. 5. The key *e* is not represented in the version because it did not appear in the key set.

**Table 2:** Mapping capabilities to operations [25]

Capability	Realization in formalism
Pure-Timeslice	Equivalent to *keyset* operation
Range-Timeslice	One *get* operation per given key
Pure-Key	One *history* operation per given key

With the given set of operations, we are able to answer all three kinds of temporal queries identified by Salzberg and Tsotras [62], as indicated in Table 2. Due to the restrictions imposed onto the *put* operation (see Definition 3), data cannot be inserted before the *now* timestamp (i.e., the history of an entry cannot be modified). Since the validity range of an entry is determined implicitly by the empty cells between changes, existing entries never need to be modified when new ones are being added. The formalization therefore fulfills all requirements stated at the beginning of this section.

### Implementation

*ChronoDB* is our implementation of the concepts presented in the previous section. It is a fully ACID compliant, process-embedded, temporal key-value store written in Java. The intended use of ChronoDB is to act as the storage backend for a graph database, which is the main driver behind numerous design and optimization choices. The full source code is freely available on GitHub under an open-source license.

#### Implementing the matrix

As the formal foundation includes the concept of a matrix with infinite dimensions, a direct implementation is not feasible. However, a Temporal Data Matrix is typically very *sparse*. Instead of storing a rigid, infinite matrix structure, we focus exclusively on the non-empty entries and expand the underlying data structure as more entries are being added.

There are various approaches for storing versioned data on disk [15, 46, 50]. We reuse existing, well-known and well-tested technology for our prototype instead of designing custom disk-level data structures. The temporal store is based on a regular B$^{+}$-Tree [61]. We make use of the implementation of B$^{+}$-Trees provided by the *TUPL*^8^ library. In order to form an actual index key from a Temporal Key, we concatenate the actual key string with the timestamp (left-padded with zeros to achieve equal length), separated by an ‘@’ character. Using the standard lexicographic ordering of strings, we receive an ordering as shown in Table 3. This implies that our B$^{+}$-Tree is ordered first by key, and then by timestamp. The advantage of this approach is that we can quickly determine the value of a given key for a given timestamp (i.e., *get* is reasonably fast), but the *keyset* (see Definition 6) is more expensive to compute.

**Table 3:** Ascending Temporal Key ordering by example [25]

Order	Temporal key	Key string	Timestamp
0	a@0123	a	123
1	a@0124	a	124
2	a@1000	a	1000
3	aa@0100	aa	100
4	b@0001	b	1
5	ba@0001	ba	1

The *put* operation appends the timestamp to the user key and then performs a regular B$^{+}$-Tree insertion. The temporal *get* operation requires retrieving the *next lower* entry with the given key and timestamp.

This is similar to regular B$^{+}$-Tree search, except that the acceptance criterion for the search in the leaf nodes is “less than or equal to” instead of “equal to”, provided that nodes are checked in descending key order. TUPL natively supports this functionality. After finding the next lower entry, we need to apply a post-processing step in order to ensure correctness of the *get* operation. Using Table 3 as an example, if we requested aa@0050 (which is not contained in the data), searching for the next-lower key produces a@1000. The key string in this temporal key (a) is different from the one which was requested (aa). In this case, we can conclude that the key aa did not exist up to the requested timestamp (50), and we return null instead of the retrieved result.

Due to the way we set up the B$^{+}$-Tree, adding a new revision to a key (or adding an entirely new key) has the same runtime complexity as inserting an entry into a regular B$^{+}$-Tree. Temporal search also has the same complexity as regular B-Tree search, which is $\mathcal {O}(\hbox {log}(n))$, where *n* is the number of entries in the tree. From the formal foundations onward, we assert by construction that our implementation will scale equally well when faced with one key and many versions, many keys with one revision each, or any distribution in between [R5]. An important property of our data structure setup is that, regardless of the versions-per-key distribution, the data structure never degenerates into a list, maintaining an access complexity of $\mathcal {O}(\hbox {log}(n))$ by means of regular B$^{+}$-Tree balancing without any need for additional algorithms.

**Fig. 6:**
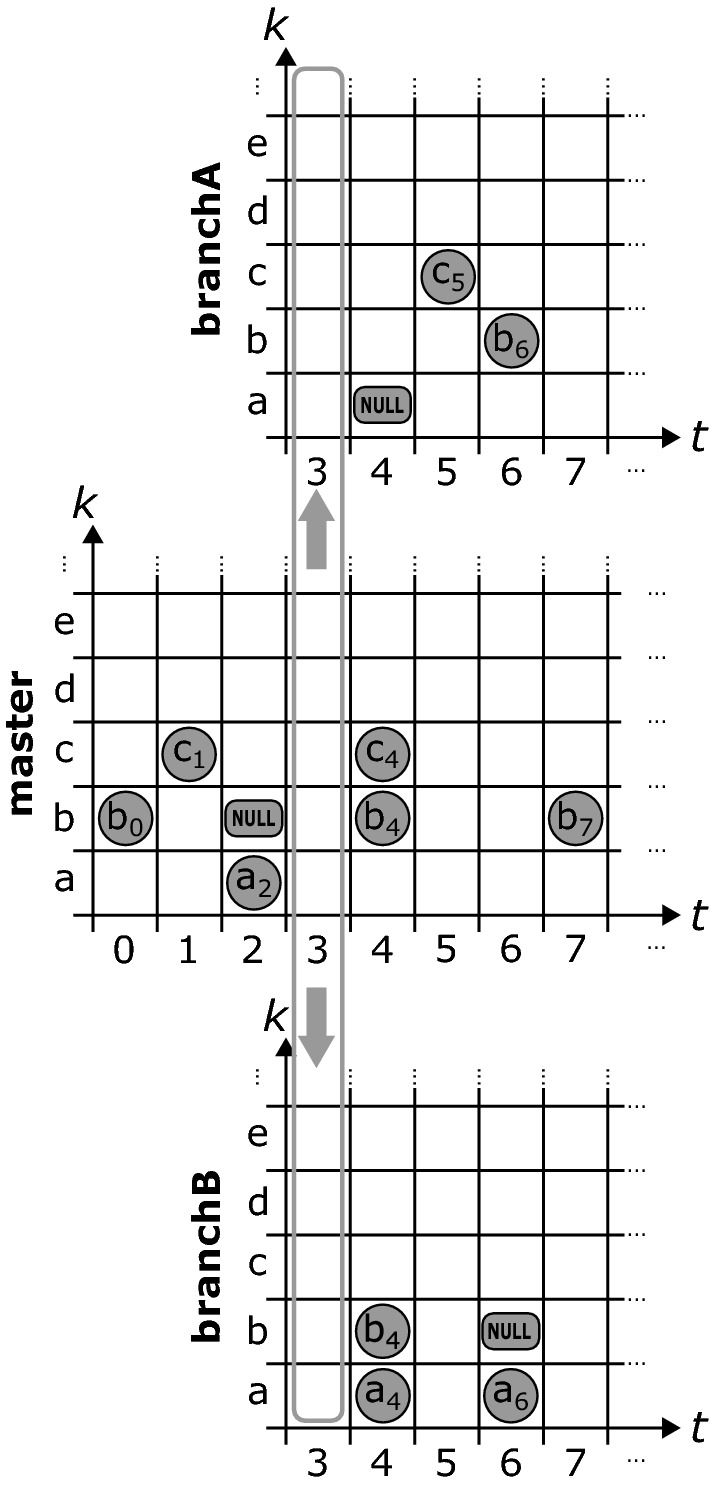
Lightweight branching concept

#### Branching

Figure 6 shows how the branching mechanism works in ChronoDB [R4]. Based on our matrix formalization, we can create branches of our history at arbitrary timestamps. To do so, we generate a new, empty matrix that will hold all *changes* applied to the branch it represents. We would like to emphasize that existing entries *are not duplicated*. We therefore create *lightweight* branches. When a *get* request arrives at the first column of a branch matrix during the search, we *redirect* the request to the matrix of the parent branch, at the branching timestamp, and continue from there. In this way, the data from the original branch (up to the branching timestamp) is still fully accessible in the child branch.

**Fig. 7:**
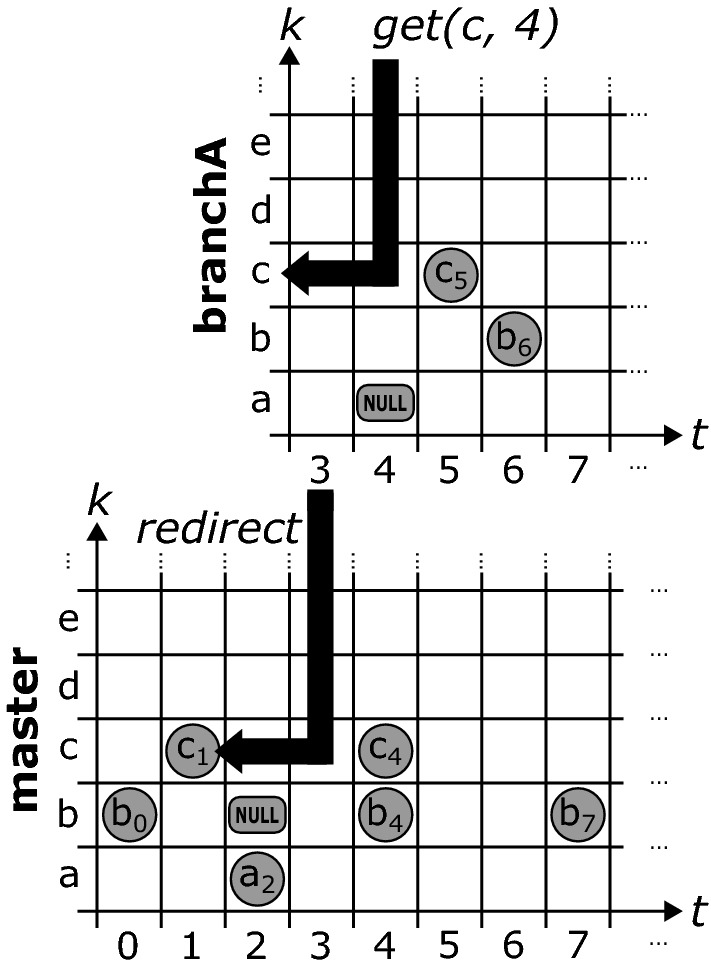
Querying data stored in branches

For example, as depicted in Fig. 7, if we want to answer a get request for key *c* on branch *branchA* and timestamp 4, we scan the row with key *c* to the left, starting at column 4. We find no entry, so we redirect the call to the origin branch (which in this case is *master*), at timestamp 3. Here, we continue left and find the value $c_{1}$ on timestamp 1. Indeed, at timestamp 4 and branch *branchA*, $c_{1}$ is still valid. However, if we issue the same original query on *master*, we would get $c_{4}$ as our result. This approach to branching can also be employed recursively in a *nested* fashion, i.e., branches can in turn have sub-branches. The primary drawback of this solution is related to the recursive “backstepping” to the origin branch during queries. For deeply nested branches, this process will introduce a considerable performance overhead, as multiple B$^{+}$-Trees (one per branch) need to be opened and queried in order to answer this request. This happens more often for branches which are very thinly populated with changes, as this increases the chances of our get request scan ending up at the initial column of the matrix without encountering an occupied cell. The operation which is affected most by branching with respect to performance is the *keySet* operation (and all other operations that rely on it), as this requires a scan on every row, leading to potentially many backstepping calls.

#### Caching

A disk access is always slow compared to an in-memory operation, even on a modern solid state drive (SSD). For that reason, nearly all database systems include some way of caching the most recent query results in main memory for later reuse. ChronoDB is no exception, but the temporal aspects demand a different approach to the caching algorithm than in regular database systems, because multiple transactions can simultaneously query the state of the stored data at different timestamps. Due to the way we constructed the Temporal Data Matrix, the chance that a given key does not change at *every* timestamp is very high. Therefore, we can potentially serve queries at many different timestamps from the same cached information by exploiting the periods in which a given key does not change its value. For the caching algorithm, we apply some of the ideas found in the work of Ramaswamy [57] in a slightly different way, adapted to in-memory processing and caching idioms.

**Fig. 8:**
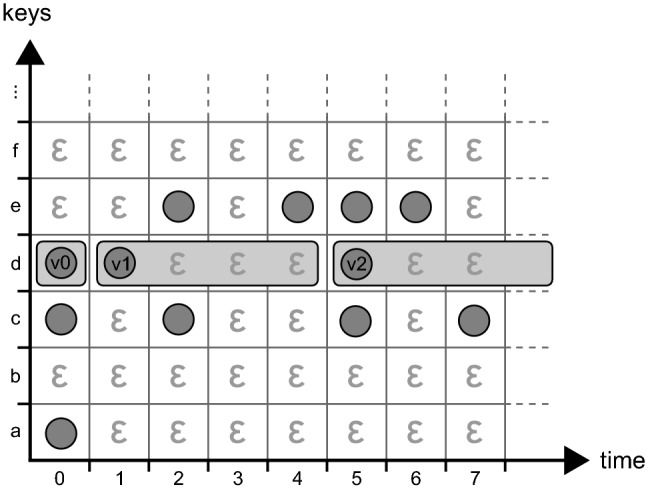
Temporal caching principle

Figure 8 displays an example for our temporal caching approach which we call *Mosaic*. When the value for a temporal key is requested and a cache miss occurs, we retrieve the value together with the validity range (indicated by gray background in the figure) from the persistent store, and add the range together with its value to the cache. Validity ranges start at the timestamp in which a key-value pair was modified (inclusive) and end at the timestamp where the next modification on that pair occurred (exclusive). For each key, the cache manages a list of time ranges called a *cache row*, and each range is associated with the value for the key in this period. As these periods never overlap, we can sort them in descending order for faster search, assuming that more recent entries are used more frequently. A cache look-up is performed by first identifying the row by the key string, followed by a linear search through the cached periods.^9^ We have a cache hit if a period containing the requested timestamp is found. When data is written to the underlying store, we need to perform a write-through in our cache, because validity ranges that have open-ended upper bounds potentially need to be shortened due to the insertion of a new value for a given key. The write-through operation is fast, because it only needs to check if the first validity range in the cache row of a given key is open-ended, as all other entries are always closed ranges. All entries in our cache (regardless of the row they belong to) share a common *least recently used* registry which allows for fast cache eviction of the least recently read entries.

In the example shown in Fig. 8, retrieving the value of key *d* at timestamp 0 would result in adding the validity range [0; 1) with value *v*0 to the cache row. This is the worst-case scenario, as the validity range only contains a single timestamp, and can consequently be used to answer queries only on that particular timestamp. Retrieving the same key at timestamps 1 through 4 yields a cache entry with a validity range of [1; 5) and value *v*1. All requests on key *d* from timestamp 1 through 4 can be answered by this cache entry. Finally, retrieving key *d* on a timestamp greater than or equal to 5 produces an open-ended validity period of $[5;\infty )$ with value *v*2, which can answer all requests on key *d* with a timestamp larger than 4, assuming that non-depicted columns are empty. If we would insert a key-value pair of $\langle d, v3\rangle$ at timestamp 10, the write-through operation would need to shorten the last validity period to [5; 10) and add a cache entry containing the period $[10;\infty )$ with value *v*3.

#### Incremental commits

Database vendors often provide specialized ways to batch-insert large amounts of data into their databases that allow for higher performance than the usage of regular transactions. ChronoDB provides a similar mechanism, with the additional challenge of keeping versioning considerations in mind along the way. Even when inserting large amounts of data into ChronoDB, we want the history to remain clean, i.e., it should not contain intermediate states where only a portion of the overall data was inserted. We therefore need to find a way to conserve RAM by writing incoming data to disk while maintaining a clean history. For this purpose, the concept of *incremental commits* was introduced in ChronoDB. This mechanism allows to mass-insert (or mass-update) data in ChronoDB by splitting it up into smaller batches while maintaining a clean history and all ACID properties for the executing transaction.

**Fig. 9:**
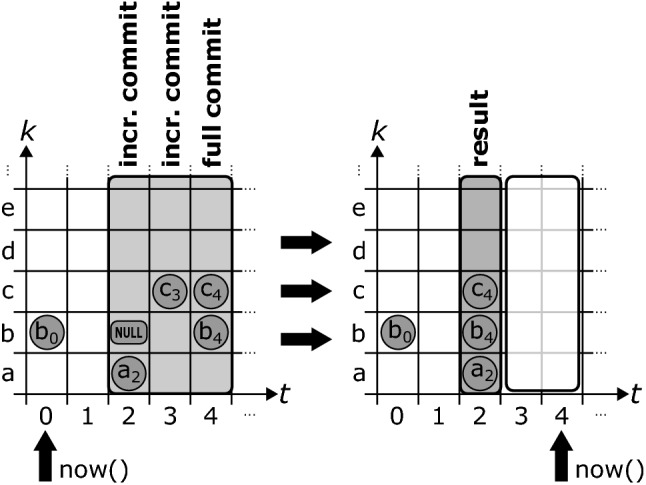
Incremental commits

Figure 9 shows how incremental commits work in ChronoDB. The process starts with a regular transaction inserting data into the database before calling commitIncremental(). This writes the first batch (timestamp 2 in Fig. 9) into the database and releases it from RAM. However, the *now* timestamp is not advanced yet. We do not allow other transactions to read these new entries, because there is still data left to insert. We proceed with the next batches of data, calling commitIncremental() after each one. After the last batch was inserted, we conclude the process with a call to commit(). This will *merge* all of our changes into one timestamp on disk. In this process, the last change to a single key is the one we keep. In the end, the timestamps between the first initial incremental commit (exclusive) to the timestamp of the final commit (inclusive) will have no changes (as shown in timestamps 3 and 4 in Fig. 9). With the final commit, we also advance the *now* timestamp of the matrix and allow all other transactions to access the newly inserted data. By delaying this step until the end of our operation, we keep the possibility to roll back our changes on disk (for example in case that the process fails) without violating the ACID properties for all other transactions. Also, if data generated by a partially complete incremental commit process is present on disk at database start-up (which occurs when the database is unexpectedly shut down during an incremental commit process), these changes can be rolled back as well, which allows incremental commit processes to have “all or nothing” semantics.

A disadvantage of this solution is that there can be only one concurrent incremental commit process on any data matrix. This process requires exclusive write access to the matrix, blocking all other (regular and incremental) commits until it is complete. However, since we only modify the head revisions and *now* does not change until the process ends, we can safely perform read operations in concurrent transactions, while an incremental commit process is taking place. Overall, incremental commits offer a way to insert large quantities of data into a single timestamp while conserving RAM without compromising ACID safety at the cost of requiring exclusive write access to the database for the entire duration of the process. These properties make them very suitable for data imports from external sources, or large scale changes that affect most of the key-value pairs stored in a matrix. This will become an important factor when we consider global model evolutions in the model repository layer [R3]. We envision incremental commits to be employed for administrative tasks which do not recur regularly, or for the initial filling of an empty database.

#### Supporting long histories

In order to create a sustainable versioning mechanism, we need to ensure that our system can support a virtually unlimited number of versions [R2, R5]. Ideally, we also should not store all data in a single file, and old files should remain untouched when new data is inserted (which is important for file-based backups). For these reasons, we must not constrain our solution to a single B-Tree. The fact that past revisions are immutable in our approach led to the decision to split the data along the time axis, resulting in a series of B-Trees. Each tree is contained in one file, which we refer to as a *chunk file*. An accompanying *meta file* specifies the time range which is covered by the chunk file. The usual policy of ChronoDB is to maximize sharing of unchanged data as much as possible. Here, we deliberately introduce data duplication in order to ensure that the initial version in each chunk is complete. This allows us to answer *get* queries within the boundaries of a single chunk, without having to navigate to the previous one. As each access to another chunk has CPU and I/O overhead, we should avoid accesses on more than one chunk to answer a basic query. Without duplication, accessing a key that has not changed for a long time could potentially lead to a linear search through the chunk chain which contradicts the requirement for scalability [R5].

**Fig. 10:**
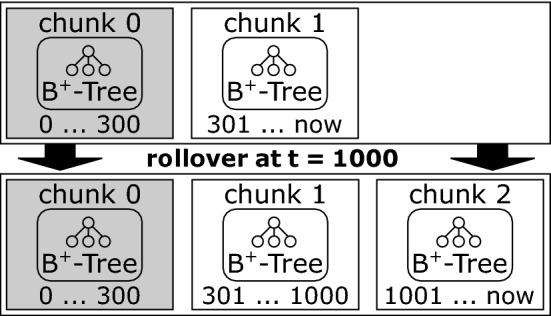
The temporal rollover process by example [26]

The algorithm for the “rollover” procedure outlined in Fig. 10 works as follows.
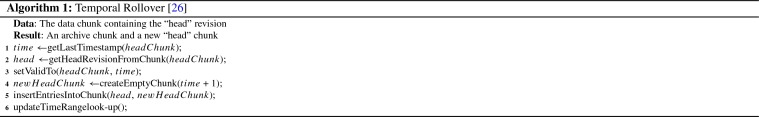


In Line 1 of Algorithm 1, we fetch the latest timestamp where a commit has occurred in our current head revision chunk. Next, we calculate the full head version of the data in Line 2. With the preparation steps complete, we set the end of the validity time range to the last commit timestamp in Line 3. This only affects the metadata, not the chunk itself. We now create a new, empty chunk in Line 4, and set the start of its validity range to the split timestamp plus one (as chunk validity ranges must not overlap). The upper bound of the new validity range is infinity. In Line 5 we copy the head version of the data into the new chunk. Finally, we update our internal look-up table in Line 6. This entire procedure only modifies the last chunk and does not touch older chunks, as indicated by the grayed-out boxes in Fig. 10.

The look-up table that is being updated in Algorithm 1 is a basic tree map which is created at start-up by reading the metadata files. For each encountered chunk, it contains an entry that maps its validity period to its chunk file. The periods are sorted in ascending order by their lower bounds, which is sufficient because overlaps in the validity ranges are not permitted. For example, after the rollover depicted in Fig. 10, the time range look-up would contain the entries shown in Table 4.

**Table 4:** Time range look-up [26]

Time range	Chunk number
$[0 \ldots 300]$	0
$[301 \ldots 1000]$	1
$[1001 \ldots \infty ]$	2

We employ a tree map specifically in our implementation for Table 4, because the purpose of this look-up is to quickly identify the correct chunk to address for an incoming request. Incoming requests have a timestamp attached, and this timestamp may occur exactly at a split, or anywhere between split timestamps. As this process is triggered very often in practice and the time range look-up map may grow quite large over time, we are considering to implement a cache based on the least-recently-used principle that contains the concrete resolved timestamp-to-chunk mappings in order to cover the common case where one particular timestamp is requested more than once in quick succession.

With this algorithm, we can support a virtually unlimited number of versions [R6] because new chunks always only contain the head revision of the previous ones, and we are always free to roll over once more should the history within the chunk become too large. We furthermore do not perform writes on old chunk files anymore, because our history is immutable. Regardless, thanks to our time range look-up, we have close to $O(\log {}n)$ access complexity to any chunk, where *n* is the number of chunks.

This algorithm is a trade-off between disk space and scalability. We introduce data duplication on disk in order to provide support for large histories. The key question that remains is *when* this process happens. We require a metric that indicates the amount of data in the current chunk that belongs to the history (as opposed to the head revision) and thus can be archived if necessary by performing a rollover. We introduce the *Head–History–Ratio* (HHR) as the primary metric for this task, which we defined as follows:

$$\begin{aligned} HHR(e, h) = {\left\{ \begin{array}{ll} e, &{} \text {if } e = h\\ \frac{h}{e-h}, &{} \text {otherwise} \end{array}\right. } \end{aligned}$$

...where *e* is the total number of entries in the chunk, and *h* is the size of the subset of entries that belong to the head revision (excluding entries that represent deletions). By dividing the number of entries in the head revision by the number of entries that belong to the history, we get a proportional notion of how much history is contained in the chunk that works for datasets of any size. It expresses how many entries we will “archive” when a rollover is executed. When new commits add new elements to the head revision, this value increases. When a commit updates existing elements in the head revision or deletes them, this value decreases. We can employ a threshold as a lower bound on this value to determine when a rollover is necessary. For example, we may choose to perform a rollover when a chunk has an HHR value of 0.2 or less. This threshold will work independently of the absolute size of the head revision. The only case where the HHR threshold is never reached is when exclusively new (i.e., never seen before) keys are added, steadily increasing the size of the head revision. However, in this case, we would not gain anything by performing a rollover, as we would have to duplicate all of those entries into the new chunk to produce a complete initial version. Therefore, the HHR metric is properly capturing this case by never reaching the threshold, thus never indicating the need for a rollover.

#### Secondary indexing

There are two kinds of secondary indices in ChronoDB. On the one hand, there are indices which are managed by ChronoDB itself (“system indices”) and on the other hand there are user-defined indices. As indicated in Table 3, the primary index for each matrix in ChronoDB has its keys ordered first by user key and then by version. In order to allow for efficient time range queries, we maintain a secondary index that is first ordered by timestamp and then by user key. Further system indices include an index for commit metadata (e.g., commit messages) that maps from timestamp to metadata, as well as auxiliary indices for branching (branch name to metadata). User-defined indices [R5] help to speed up queries that request entries based on their contents (rather than their primary key). An example for such a query is *find all persons where the first name is ’Eva’*. Since ChronoDB stores arbitrary Java objects, we require a method to *extract* the desired property value to index from the object. This is accomplished by defining a ChronoIndexer interface. It defines the index(Object) method that, given an input object, returns the value that should be put on the secondary index. Each indexer is associated with a name. That name is later used in a query to refer to this index. The associated query language provides support for a number of string matching techniques (equals, contains, starts with, regular expression...), numeric matching (greater than, less than or equal to...) as well as Boolean operators (and, or, not). The query engine also performs optimizations such as double negation elimination. Overall, this query language is certainly less expressive than other languages such as SQL. Since ChronoDB is intended to be used as a storage engine and embedded in a database frontend (e.g., a graph database), these queries will only be used internally for index scans while more sophisticated expressions are managed by the database frontend. Therefore, this minimalistic Java-embedded DSL has proven to be sufficient. An essential drawback of this query mechanism is that the number of properties available for querying is determined by the available secondary indices. In other words, if there is no secondary index for a property, that property cannot be used for filtering. This is due to ChronoDB being agnostic to the Java objects it is storing. In absence of a ChronoIndexer, it has no way of extracting a value for an arbitrary request property from the object. This is a common approach in database systems: without a matching secondary index, queries require a linear scan of the entire data store. When using a database frontend, this distinction is blurred, and the difference between an index query and a non-index query is only noticeable in how long it takes to produce the result set.

In contrast to the primary index, entries in the secondary index are allowed to have *non-unique* keys. For example, if we index the “name” attribute, then there may be more than one entry where the name is set to “John”. We therefore require a different approach than the temporal data matrices employed for the primary index. Inspired by the work of Ramaswamy et al. [57], we make use of explicit time windows. Non-unique indices in versioned contexts are special cases of the general *interval stabbing problem* [31].

**Table 5:** Secondary indexing in ChronoDB

#	Index	Branch	Keyspace	Key	Value	From	To
1	name	master	default	e1	“john”	1234	$\infty$
2	name	master	default	e2	“john”	1234	5678
3	name	master	default	e3	“john”	1234	7890
4	name	master	default	e2	“jack”	5678	$\infty$

Table 5 shows an example of a secondary index. As such a table can hold all entries for all indices, we store the index for a particular entry in the “index” column. The branch, keyspace and key columns describe the location of the entry in the primary index. The “value” column contains the value that was extracted by the ChronoIndexer. “From” and “To” express the time window in which a given row is valid. Any entry that is newly inserted into this table initially has its “To” value set to infinity (i.e., it is valid for an unlimited amount of time). When the corresponding entry in the primary index changes, the “To” value is updated accordingly. All other columns are effectively immutable.

In the concrete example shown in Table 5, we insert three key-value pairs (with keys *e*1, *e*2 and *e*3) at timestamp 1234. Our indexer extracts the value for the “name” index, which is “john” for all three values. The “To” column is set to infinity for all three entries. Querying the secondary index at that timestamp for all entries where “name” is equal to “john” would therefore return the set containing *e*1, *e*2 and *e*3. At timestamp 5678, we update the value associated with key *e*2 such that the indexer now yields the value “jack”. We therefore need to terminate the previous entry (row #2) by setting the “To” value to 5678 (upper bounds are exclusive), and inserting a new entry that starts at 5678, has the value “jack” and an initial “To” value of infinity. Finally, we delete the key *e*3 in our primary index at timestamp 7890. In our secondary index, this means that we have to limit the “To” value of row #3 to 7890. Since we have no new value due to the deletion, no additional entries need to be added.

This tabular structure can now be queried using well-known techniques also employed by SQL. For usual queries, the branch and index is fixed, the value is specified as a search string and a condition (e.g., “starts with [jo]”) and we know the timestamp for which the query should be evaluated. We process the timestamp by searching only for entries where

$$\begin{aligned} From \le timestamp < To \end{aligned}$$

...in addition to the conditions specified for the other columns. Selecting only the documents for a given branch is more challenging, as we need to traverse the origin branches upwards until we arrive at the master branch, performing one subquery for each branch along the way and merging the intermediate results accordingly.

**Fig. 11:**
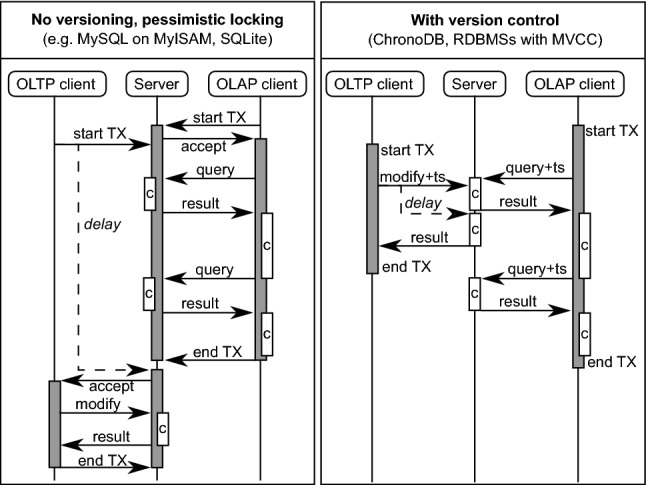
Transaction control with and without versioning [25]

#### Transaction control

Consistency and reliability are two major goals in ChronoDB. It offers full ACID transactions with the highest possible read isolation level (*serializable*, see [38]). Figure 11 shows an example with two sequence diagrams with identical transaction schedules. A database server is communicating with an Online Analytics Processing (OLAP [10]) client that owns a long-running transaction (indicated by gray bars). The process involves messages (arrows) sending queries with timestamps and computation times (blocks labeled with “c”) on both machines. A regular Online Transaction Processing (OLTP) client wants to make changes to the data which is analyzed by the OLAP client. The left figure shows what happens in a non-versioned scenario with pessimistic locking. The server needs to lock the relevant contents of the database for the entire duration of the OLAP transaction, otherwise we risk inconsistencies due to the incoming OLTP update. We need to delay the OLTP client until the OLAP client closes the transaction. Modern databases use optimistic locking and data duplication techniques (e.g., MVCC [6]) to mitigate this issue, but the core problem remains: the server needs to *dedicate resources* (e.g., locks, RAM...) to client transactions over their entire lifetime. With versioning, the OLAP client sends the query plus the request timestamp to the server. This is a self-contained request; no additional information or resources are needed on the server, and yet the OLAP client achieves full isolation over the entire duration of the transaction, because it always requests the same timestamp. While the OLAP client is processing the results, the server can safely allow the modifications of the OLTP client, because it is guaranteed that any modification will only *append* a new version to the history. The data at timestamp on which the OLAP client is working is immutable. Client-side transactions act as containers for transient change sets and metadata, most notably the timestamp and branch name on which the transaction is working. Security considerations aside, transactions can be created (and disposed) without involving the server. An important problem that remains is how to handle situations in which two concurrent OLTP transactions attempt to change the same key-value pair. ChronoDB allows to select from several conflict handling modes (e.g., reject, last writer wins) or to provide a custom conflict resolver implementation.

## Solution part II: ChronoGraph

ChronoGraph is our versioned graph database which is built on top of ChronoDB. ChronoGraph implements the Apache TinkerPop standard, the de-facto standard interface for graph databases. We first provide a high-level overview over TinkerPop; then, we focus on the concepts of ChronoGraph itself.

**Fig. 12:**
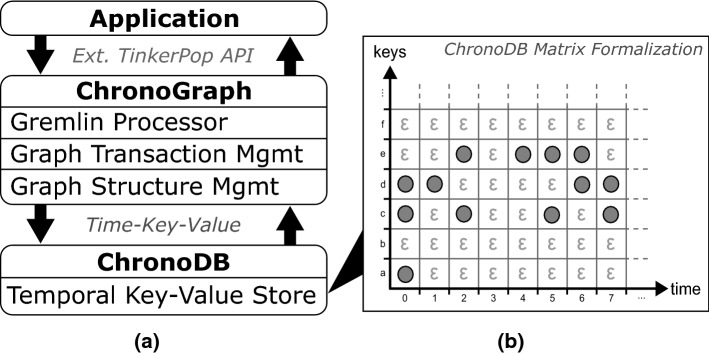
ChronoGraph architecture [27]

### Apache TinkerPop

The *TinkerPop* framework is the *de-facto* standard interface between applications and graph databases. Its main purpose is to allow application developers to exchange a graph database implementation with another one without altering the application source code that accesses the database. The TinkerPop standard is designed in a modular fashion. The core module is the *property graph API* [58] which specifies the Java interfaces for vertices, edges, properties and other structural elements.

In a property graph, each vertex and edge can have properties which are expressed as key–value pairs. According to the standard, each vertex must have an identifier which is unique among all vertices, and the same is true for edges. In practice, database vendors often recommend to use identifiers which are globally unique in the database. Furthermore, in addition to the unique ID and the user-defined properties, each vertex and edge has a label, which is defined to be a single-valued string that is intended to be used for categorization purposes. All user-defined properties are untyped by definition, i.e., no restriction is imposed on which values a user-defined property may have. However, some graph database vendors such as Titan DB^10^ and OrientDB^11^ offer the possibility to define a schema which is evaluated at runtime. The only unsupported value for any user-defined property is the null value. Instead of assigning null to a property, it is recommended to delete the property on the target graph element entirely.

Another module in the TinkerPop standard is the graph query language *Gremlin*. In contrast to the property graph API, which is only a specification, Gremlin comes with a default implementation that is built upon the property graph API interfaces. This implementation also includes a number of built-in query optimization strategies. Other modules include a standard *test suite* for TinkerPop vendors, and a generic server framework for graph databases called *Gremlin Server*.

### ChronoGraph architecture

Our open-source project ChronoGraph provides a fully TinkerPop-compliant graph database implementation with additional versioning capabilities. In order to achieve this goal, we employ a layered architecture as outlined in Fig. 12a. In the remainder of this section, we provide an overview of this architecture in a bottom-up fashion.

The bottom layer of the architecture is a versioned key-value store, i.e., a system capable of working with *time–key–value* tuples as opposed to plain key–value pairs in regular key-value stores. For the implementation of ChronoGraph, we use ChronoDB, as introduced in Sect. 4.

ChronoGraph itself consists of three major components. The first component is the *graph structure* management. It is responsible for managing the individual vertices and edges that form the graph, as well as their referential integrity [R9]. As the underlying storage mechanism is a key-value store, the graph structure management layer also performs the partitioning of the graph into key-value pairs and the conversion between the two formats. We present the technical details of this format in Sect. 5.3. The second component is the *transaction management*. The key concept here is that each graph transaction is associated with a timestamp on which it operates. Inside a transaction, any read request for graph content will be executed on the underlying storage with the transaction timestamp. ChronoGraph supports full ACID transactions [R6] with the highest possible isolation level (“serializable”, also known as “snapshot isolation”, as defined in the SQL Standard [38]). The underlying versioning system acts as an enabling technology for this highest level of transaction isolation, because any given version of the graph, once written to disk, is effectively immutable. All mutating operations are stored in the transaction until it is committed, which in turn produces a new version of the graph, with a new timestamp associated with it. Due to this mode of operation, we do not only achieve repeatable reads, but also provide effective protection from phantom reads, which are a common problem in concurrent graph computing. The third and final component is the *query processor* itself which accepts and executes Gremlin queries on the graph system. As each graph transaction is bound to a branch and timestamp, the query language (Gremlin) remains agnostic of both the branch and the timestamp, which allows the execution of any query on any desired timestamp and branch [R8].

The application communicates with ChronoGraph by using the regular TinkerPop API, with additional extensions specific to versioning. The versioning itself is entirely transparent to the application to the extent where ChronoGraph can be used as a drop-in replacement for any other TinkerPop 3.x compliant implementation. The application is able to make use of the versioning capabilities via additional methods, but their usage is entirely optional and not required during regular operation that does not involve history analysis.

### Data layout

In order to store graph data in our Temporal Key–Value Store, we first need to disassemble the graph into partitions that can be serialized as values and be addressed by keys. Then, we need to persist these pairs in the store. We will first discuss how we disassemble the graph, followed by an overview of the concrete key–value format and how versioning affects this process.

#### Partitioning: the star graph format

Like many other popular graph databases, e.g., Titan DB, we rely on the *Star Graph* partitioning in order to disassemble the graph into manageable pieces.

**Fig. 13:**
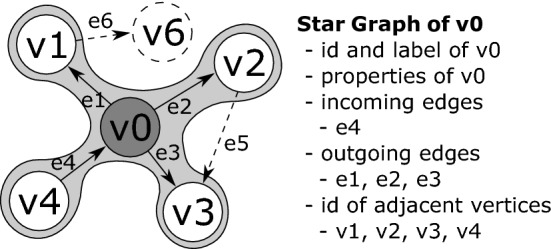
Star Graph partitioning by example

Figure 13 shows an example of a star graph. A star graph is a subset of the elements of a full graph that is calculated given an origin vertex, in this case *v*0. The star graph contains all properties of the vertex, including the *id* and the *label*, as well as all incoming and outgoing edges (including their *label*, *id* and properties). All adjacent vertices of the origin vertex are represented in the star graph by their *id*s. Their attributes and remaining edges (indicated by dashed lines in Fig. 13) are not contained in the star graph of *v*0. This partitioning was chosen due to its ability to reconstruct the entire graph from disk without duplicating entire vertices or attribute values. Furthermore, it is suitable for lazy loading of individual vertices, as only the immediate neighborhood of a vertex needs to be loaded to reconstruct it from disk.

#### Key–value layout

Starting from a star graph partitioning, we design our key–value layout. Since all graph elements in TinkerPop are mutable by definition and our persistent graph versions have to be immutable, we perform a bijective mapping step before persisting an element. We refer to the persistent, immutable version as a *Record*, and there is one type of record for each structural element in the TinkerPop API. For example, the mutable *Vertex* element is mapped to an immutable *VertexRecord*. A beneficial side-effect of this approach is that we hereby gain control over the persistent format, and can evolve and adapt each side of the mapping individually if needed. Table 6 shows the contents of the most important record types.

**Table 6:** TinkerPop API to Record Mapping [27]

TinkerPop	Record	Record contents
Vertex	VertexRecord	id, label,
		PropertyKey $\rightarrow$ PropertyRecord
		In: EdgeLabel $\rightarrow$ EdgeTargetRecord
		Out: EdgeLabel $\rightarrow$ EdgeTargetRecord
Edge	EdgeRecord	id, label,
		PropertyKey $\rightarrow$ PropertyRecord
		id of InVertex, id of OutVertex
Property	PropertyRecord	PropertyKey, PropertyValue
—	EdgeTargetRecord	id of edge, id of other-end Vertex

In Table 6, all *id* and *label* elements, as well as all *PropertyKey*s, are of type String. The *PropertyValue* in the *PropertyRecord* is assumed to be in byte array form. An arrow in the table indicates that the record contains a *mapping*, usually implemented with a regular hash map. An element that deserves special attention is the *EdgeTargetRecord* that does not exist in the TinkerPop API. Traversing from one vertex to another via an edge label is a very common task in a graph query. In a naive mapping, we would traverse from a vertex to an adjacent edge and load it, find the id of the vertex at the other end, and then resolve the target vertex. This involves two steps where we need to resolve an element by ID from disk. However, we cannot store all edge information directly in a *VertexRecord*, because this would involve duplication of all edge properties on the other-end vertex. We overcome this issue by introducing an additional record type. The *EdgeTargetRecord* stores the id of the edge and the id of the vertex that resides at the “other end” of the edge. In this way, we can achieve basic vertex-to-vertex traversal in one step. At the same time, we minimize data duplication and can support edge queries (e.g., g.traversal().E() in TinkerPop), since we have the full *EdgeRecord*s as standalone elements. A disadvantage of this solution is the fact that we still need to do two resolution steps for any query that steps from vertex to vertex and has a condition on a *property* of the edge in between. This trade-off is common for graph databases, and we share it with many others, e.g., Neo4j. We will discuss this with a concrete example in Sect. 5.4.

For each record type, we create a *keyspace* in the underlying key–value store. We serialize the record elements into a binary sequence. This binary sequence serves as the value for the key–value pairs and the id of the element is used as the corresponding key. The type of record indicates which keyspace to use, completing the mapping to the key–value format. The inverse mapping involves the same steps: given an element ID and type, we resolve the key–value pair from the appropriate keyspace by performing a key look-up using the ID. Then, we deserialize the binary sequence, and apply our bijective element-to-record mapping in the inverse direction. When loading a vertex, the properties of the incoming and outgoing edges will be loaded *lazily*, because the EdgeTargetRecord does not contain this information and loading edge properties immediately would therefore require an additional look-up. The same logic applies to resolving the other-end vertices of EdgeTargetRecords, allowing for a lazy (and therefore efficient and RAM-conserving) solution.

**Fig. 14:**
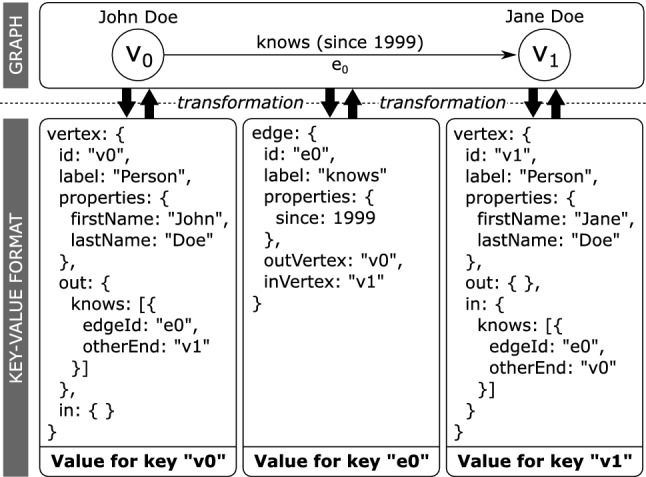
Mapping a graph to key-value format

Figure 14 shows an example for the translation process between the Graph format and the Key-Value-Store format. In this example, we express the fact “John Doe knows Jane Doe since 1999” in a property graph format. Each graph element is transformed into an entry in the key–value store. In the example, we use a JSON-like syntax; our actual implementation employs a binary serialization format. Please note that the presented value structures correspond to the schema for records presented in Table 6.

### Versioning concept

When discussing the mapping from the TinkerPop structure to the underlying key–value store in Sect. 5.3, we did not touch the topic of versioning. This is due to the fact that our key–value store ChronoDB is performing the versioning on its own. The graph structure does not need to be aware of this process. We still achieve a fully versioned graph, an immutable history and a very high degree of sharing of common (unchanged) data between revisions. This is accomplished by attaching a fixed *timestamp* to every *graph transaction*. This timestamp is always the same as in the underlying ChronoDB transaction. When reading graph data, at some point in the resolution process we perform a *get(...)* call in the underlying key–value store, resolving an element (e.g., a vertex) by ID. At this point, ChronoDB uses the timestamp attached to the transaction to perform the temporal resolution. This will return the value of the given key, at the specified timestamp.

**Fig. 15:**
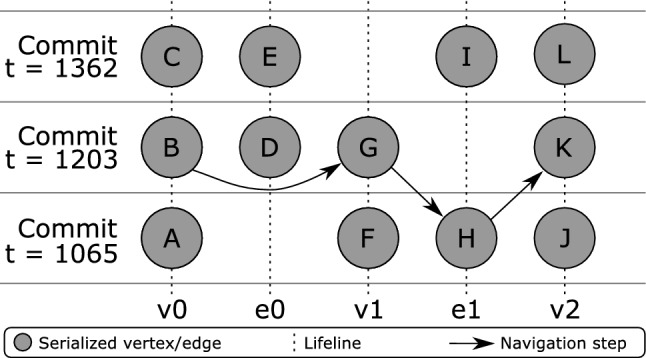
Example: Navigating in a graph version

In order to illustrate this process, we consider the example in Fig. 15. We open a transaction at timestamp 1234 and execute the following Gremlin query:

$$\begin{aligned}&{\texttt {V("v0").out("e0").outE("e1")}}\\&\quad {\texttt {.has("p", "x").inV()}} \end{aligned}$$

Translated into natural language, this query:

1. starts at a given vertex (*v0*),
2. navigates along the outgoing edge labeled as *e0* to the vertex at the other end of the edge,
3. from there navigates to the outgoing edge labeled as *e1*,
4. checks that the edge has a property *p* which is set to value *x*,
5. and finally navigates to the target vertex of that edge.

We start the execution of this query by resolving the vertex *v0* from the database. Since our transaction uses timestamp 1234, ChronoDB will look up the temporal key *v0@1234*, and return the value labeled as *B* in Fig. 15.^12^ Value *A* is not visible because it was overwritten by *B* at timestamp 1203, and value *C* is also not visible because it was written *after* our transaction timestamp. Next, we navigate the outgoing edge labeled as *e0*. Our store does contain information on that edge, but since the query does not depend on any of its properties, we use the *EdgeTargetRecord* stored in *B* and directly navigate to *v1*. We therefore ask ChronoDB for the value associated with temporal key *v1@1234*, and receive value *G*. For the next query step, we have a condition on the outgoing edge *e1*. Our EdgeTargetRecord in value *G* does not contain enough information to evaluate the condition; hence, we need resolve the edge from the store. Querying the temporal key *e1@1234* will return the value *H*, which is shared with the previous version because it was not changed since then. After evaluating the condition that the property “p” on edge version *H* is indeed set to the value “x” (as specified in the query), we continue our navigation by resolving the target of *e1*, which is *v2*. The temporal key *v2@1234* will result in the value *K* being returned.

Note that this final navigation step starts at an element that was reused from the commit at timestamp 1065 and ends at the state of *v2* that was produced by the commit at timestamp 1203. This is possible because graph elements refer to each other by ID, but these references do not include the branch or timestamp. This information is injected from the transaction at hand, allowing for this kind of navigation and data reuse. This is a major step toward fulfilling requirement [R8]. As ChronoDB offers logarithmic access time to any key-value pair on any version, this is also in line with requirement [R5].

### TinkerPop compatibility and extensions

The Apache TinkerPop API is the *de-facto* standard interface between graph databases and applications built on top of them. We therefore want ChronoGraph to implement and be fully compliant to this interface as well. However, in order to provide our additional functionality, we need to extend the default API at several points. There are two parts to this challenge. The first part is *compliance* with the existing TinkerPop API, the second part is the *extension* of this API in order to allow access to new functionality. In the following sections, we will discuss these points in more detail.

#### TinkerPop API compliance

As we described in Sects. 5.3 and 5.4, our versioning approach is entirely transparent to the user. This eases the achievement of compliance to the default TinkerPop API. The key aspect that we need to ensure is that every transaction receives a proper timestamp when the regular transaction opening method is invoked. In a non-versioned database, there is no decision to make at this point, because there is only one graph in a single state. The logical choice for a versioned graph database is to return a transaction on the current *head* revision, i.e., the timestamp of the transaction is set to the timestamp of the latest commit. This aligns well with the default TinkerPop transaction semantics—a new transaction *t*1 should see all changes performed by other transactions that were committed before *t*1 was opened. When a commit occurs, the changes are always applied to the head revision, regardless of the timestamp at hand, because history states are immutable in our implementation in order to preserve traceability of changes. As the remainder of our graph database, in particular the implementation of the query language Gremlin, is unaware of the versioning process, there is no need for further specialized efforts to align versioning with the TinkerPop API.

We employ the TinkerPop *Structure Standard Suite*, consisting of more than 700 automated JUnit tests, in order to assert compliance with the TinkerPop API itself. This test suite is set up to scan the declared *Graph Features* (i.e., optional parts of the API), and enable or disable individual tests based on these features. With the exception of *Multi-Properties*^13^ and the *Graph Computer*,^14^ we currently support all optional TinkerPop API features, which results in 533 tests to be executed. We had to manually disable 8 of those remaining test cases due to problems within the test suite, primarily due to I/O errors related to illegal file names on our Windows-based development system. The remaining 525 tests all pass on our API implementation.

#### TinkerPop extensions

Having asserted conformance to the TinkerPop API, we created custom extensions that give access to the features unique to ChronoGraph. As the query language Gremlin itself remains completely untouched in our case, and the graph structure (e.g., Vertex and Edge classes) is unaware of the versioning process (as indicated in Sect. 5.4), we are left with one possible extension point, which is the Graph interface itself. In order to offer queries access to timestamps other than the head revision, we need to add a method to open a transaction on a user-provided timestamp. By default, a transaction in TinkerPop on a Graph instance g is opened without parameters. We expand the transaction class by adding several overrides which accept the desired target branch and version. Using these additional overrides, the user can decide the java.util.Date or java.lang.Long timestamp on which the transaction should be based, as well as the branch to operate on. This small change of adding an additional time argument is all it takes for the user to make full use of the time travel feature, the entire remainder of the TinkerPop API, including the structure elements and the Gremlin query language, behave as defined in the standard. Opening a transaction without parameters defaults to opening a transaction on the latest version of the master branch, which is also in line with the TinkerPop API specification.

In order to provide access to the history of a single Vertex or Edge, we added explicit query methods to our Graph implementation. These methods allow access to the history of any given edge or vertex. The history is expressed by an Iterator over the change timestamps of the element in question, i.e., whenever a commit changed the element, its timestamp will appear in the values returned by the iterator. The user of the API can then use any of these timestamps as an argument to g.tx().open(...) in order to retrieve the state of the element at the desired point in time. The implementation of the history methods delegate the call directly to the underlying ChronoDB, which retrieves the history of the key–value pair associated with the ID of the given graph element. This history is extracted from the primary index, which is first sorted by key (which is known in both scenarios) and then by timestamp. This ordering allows the two history operations to be very efficient as only element ID requires a look-up in logarithmic time, followed by backwards iteration over the primary index (i.e., iteration over change timestamps) until a different ID is encountered (c.f. Table 3).

The final requirement with respect to versioning capabilities is the demand for an operation that lists all changes within a given time range, regardless of the affected elements. In order to meet this requirement, we added another pair of methods to our Graph implementation. These methods (one for vertices, one for edges) accept time ranges and grant access to iterators that return *TemporalKey*s. These keys are pairs of actual element identifiers and change timestamps. Just as their element-specific counterparts, it is intended that these timestamps are used for opening transactions on them in order to inspect the graph state. Combined calls to next() on it1 and it2 will yield the complete list of changes upon iterator exhaustion. Analogous to their element-specific siblings, these methods redirect directly to the underlying ChronoDB instance, where a secondary temporal index is maintained that is first ordered by timestamp and then by key. This secondary index is constructed per keyspace. Since vertices and edges reside in disjoint keyspaces, these two operations do not require further filtering and can make direct use of the secondary temporal index.

### Transaction semantics

The Apache TinkerPop API is currently available in its third version. It evolved alongside its implementations, which range from local graphs (e.g., the in-memory reference implementation TinkerGraph) to highly distributed systems (e.g., Titan DB). Due to this diversity, the requirements toward transaction semantics, in particular behavior under concurrent access [R6], are specified very loosely in TinkerPop itself. For example, when iterating over the outgoing edges of a vertex, TinkerPop only specifies that the iteration itself should never return a null value and should never throw a ConcurrentModificationException, but details regarding the visibility of changes made by other, concurrent transactions are unspecified.

Since the reference implementation TinkerGraph, which is provided alongside the API, does not support transactions,^15^ we had to design the transaction semantics by ourselves. When we implemented ChronoDB, we envisioned it to be a system suitable for storing data for analysis purposes, therefore the consistency of a view and the contained data is paramount. As all stored versions are effectively immutable, we chose to implement a full ACID transaction model in ChronoDB with the highest possible isolation level (“Serializable” [38]). As ChronoGraph is based on ChronoDB, it follows the same transaction model. To the best of our knowledge, ChronoGraph is currently the only implementation of the TinkerPop API v3.x that is full ACID in the strict sense, as many others opt for *repeatable reads* isolation (e.g., OrientDB), while ChronoGraph supports *snapshot* isolation. A proposal for snapshot isolation for Neo4j was published recently [55], but it is not part of the official version. Graph databases without ACID transactions and snapshot isolation often suffer from issues like *Ghost Vertices*^16^ or *Half Edges*^17^ which can cause inconsistent query results and are very difficult to deal with as an application developer. These artifacts are negative side-effects of improper transaction isolation, and application developers have to employ techniques such as soft deletes (i.e., the addition of “deleted” flags instead of true element deletions) in order to avoid them. As ChronoGraph adheres to the ACID properties, these inconsistencies can not appear by design.

### Functionality and usage implications

Our implementation is a stark contrast to existing solutions. We implement the versioning process at a lower level, in the generic temporal key–value store ChronoDB. This store is aware of the semantics of the versioning process, and is capable of solving the problem of long histories [26] (c.f. Sect. 4.2), unlike the previously mentioned solutions. There are no additional mapping steps required in order to achieve graph versioning, in fact our graph to key-value mapping is very similar to the algorithm employed by Titan DB. In particular, no additional auxiliary graph elements are introduced for the purpose of versioning. To the end user, the versioning process is completely transparent, as our implementation is fully compliant with the standard TinkerPop API for non-versioned graphs. There is no need for translating one graph query into another in order to run it on a different version of the graph. A developer familiar with the TinkerPop API can start using ChronoGraph without any particular knowledge about its versioned nature. By offering additional methods, which are very much in line with the intentions of the TinkerPop API, we grant access to the versioning-related features. Additionally, ChronoGraph is fully ACID compliant with snapshot isolation for concurrent transactions, preventing common artifacts that arise in other, non-ACID graph databases, such as ghost vertices and half edges. Our solution is strongly based on immutability of existing versions, which aids in preserving traceability of changes and allows extensive sharing of data that remained unchanged between revisions.

### Conflict resolution

In case of concurrent write transactions, the versioning engine is sometimes faced with conflicting commits. This situation occurs when two transactions simultaneously intend to modify the very same graph element. In this section, we describe our current conflict resolution approach.

The conflict resolution algorithm implemented in ChronoGraph differentiates between addition and removal of entire graph elements on the one hand and property value changes on the other hand. Additions of graph elements can never cause a true conflict: even if two concurrent transactions add a vertex or edge with the same new identifier (which is highly unlikely, since we employ universally unique identifiers), then the resulting state in both transactions is identical: the new vertex or edge exists. They may still differ in their properties, which we consider at a later stage.

When either side of the conflict is a graph element deletion, we are faced with two options: either we undo the deletion to apply the changes from the other side of the conflict, or we retain the deletion and discard the other changes. In our current implementation, the removal of a graph element always takes precedence over any other conflicting modification on this element. This may cause the loss of property changes on the deleted element in the concurrent transaction. However, the alternative of “undeleting” the graph element is even more undesirable. In particular if the deleted element was a vertex, then its adjacent edges have also been deleted, which would result in a completely isolated vertex if we chose to undelete it. Isolated vertices are no problem for the storage engine, but they don’t add much value to the semantics of the graph, as they will never contribute to the results of traversal queries (since they are not connected to any other element).

In case of a conflict on property values, it is important to know that ChronoGraph tracks all modifications on vertex and edge properties individually. This means that the conflict resolution algorithm has access to the information whether or not any given vertex property or edge property has been modified within the transaction. For example, if two concurrent transactions perform a commit on the same vertex, and one of them sets the firstname property to John, while the other sets the lastname property to Doe, then the conflict is resolved by accepting both changes (the resulting vertex will have a firstname of John and a lastname of Doe). Only if both transactions modify the same property on the same graph element, then a true conflict occurs. Here, we employ the same strategy as for graph elements: if either side of the conflict is a deletion, the deletion wins. If neither side is a deletion, then the last writer wins. For example, if one transaction sets firstname to John and a concurrent transaction sets firstname to Jack on the same graph element, then the conflict is resolved by using the firstname value from the transaction which was committed later in time.

### Limitations and drawbacks

Our approach is tailored toward the use case of having a *versioned* graph (as opposed to a *temporal* graph), which entails that queries on a *single* timestamp are the prevalent form of read access. Even though we support additional auxiliary methods for traversing the history of a single vertex or edge, and listing all changes within a given time range, our approach is far less suitable for use cases with an emphasis on temporal analysis that require time range queries, or detection of patterns on the time axis (as in graph stream analysis [47, 56]). For example, answering the question “Which elements often change together?”, while possible in our solution, can not be implemented in an efficient way that does not require linear scanning through the commit logs. Another example would be the query “List all vertices that have ever been adjacent to a given one”, which would again involve linear iteration in our solution. In general, our graph is a TinkerPop implementation and therefore optimized with the traversal language *Gremlin* in mind. As such, it does not lend itself as well to declarative, pattern-driven search approaches like *Cypher* as a dedicated Cypher graph implementation (e.g., Neo4j) would do.

**Fig. 16:**
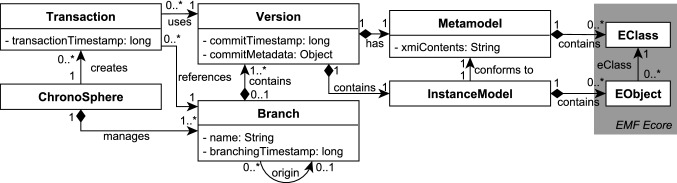
Conceptual ChronoSphere metamodel [28]

We are currently also not offering any means for distributing the graph among multiple machines (see Sect. 10 for details). This limits the scale of our graph to sizes manageable within the physical memory and computing resource restrictions of a single machine. An essential drawback of our solution is that, due to the versioned nature of our data, we cannot rely as much on dictionaries with $\mathcal {O}(1)$ access times (e.g., Hash Maps) as regular general-purpose graph databases, because of the temporal resolution steps that happen on every navigation. Those steps have a complexity of $\mathcal {O}(\log {}(n))$, which also limits the scalability of our graph.

Finally, we have to acknowledge the fact that ChronoGraph is an ongoing work-in-progress research project, therefore numerous optimization possibilities have not been exploited yet. For a detailed evaluation of ChronoGraph, we refer the interested reader to our previous work [27].

## Solution part III: ChronoSphere

ChronoSphere is our novel open-source graph-based EMF model repository. It provides a wide variety of features known from other solutions, such as querying, persistence, versioning and branching, and furthermore supports unique features such as metamodel evolution and snapshot-level transaction isolation. ChronoSphere does not assume the presence of a runtime environment such as OSGi,^18^ but can be integrated into such frameworks if required. The software is distributed via standard Maven repositories,^19^ which makes it easily accessible for a wide range of modern dependency management tools such as Gradle, Apache Ivy or Apache Maven. ChronoSphere is implemented in pure Java, which allows it to run in any environment compatible with the Java 8 SE standard. This includes a broad spectrum of scenarios, from single-user desktop applications to highly concurrent enterprise-level server back-ends. The only notable exception where ChronoSphere cannot be used are JVM implementations that do not support Java reflection (e.g., Android devices) which is required for serialization and deserialization of objects in the lower levels of ChronoDB. As ChronoSphere provides its own data store and has no dependencies to an external database, it can be easily embedded into any EMF-based application.

A conceptual metamodel of ChronoSphere is shown in Fig. 16. A ChronoSphere instance manages a number of named Branches (with *master* as the predefined one) [R4], and each Branch refers to its origin (recursively). Each Branch contains any number of Versions [R8], which in turn contain a user-defined (Ecore-based) Metamodel and an InstanceModel, which is a collection of EObjects that adhere to the EClasses in the Metamodel. A ChronoSphere instance can then create Transactions [R6] on a given Version by starting them on a transactionTimestamp, which is usually obtained from a user-provided java.util.Date. This is a fairly common setup for versioning- and branching-enabled model repositories. A detail deserving special attention is the fact that a Version and a Metamodel are bound to each other in a one-to-one relationship. This is a requirement for metamodel evolution [R3], which we will discuss in Sect. 6.4.

### Graph layout

In order to store EMF metamodels and their corresponding instance models in our graph database, we need to define a bijective transformation for each element between its EMF (in-memory) representation and its (persistent) graph representation. Our approach to this task is inspired by Neo4EMF [4] which uses a similar mapping.

**Fig. 17:**
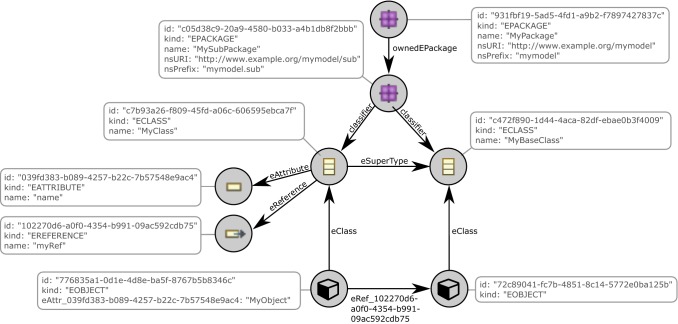
Model-to-graph mapping by example (simplified)

Figure 17 shows a small example for our model-to-graph mapping. Please note that this example is not complete; several properties were omitted for visual clarity. As outlined earlier, we store the Ecore metamodel (EPackages, EClasses, ...) together with the actual instance model (EObjects) in the *same* graph in order to support metamodel evolution and model versioning at the same time. The two vertices at the top represent two EPackages, with “MySubPackage” being owned by “MyPackage”. The metamodel also contains two EClasses, one of which has an EAttribute and an EReference attached. Note that, in contrast to regular Ecore, we attach unique identifiers to every element in the meta- and instance model. This allows for easier object comparison in cases where an element was loaded more than once from the graph.

The two vertices with box icons represent actual EObjects. Each EObject vertex is connected with an edge labeled as “eClass” to the vertex in the metamodel that represents the EClass of the EObject. References are represented as edges as well. They use the unique ID of the EReference to which they belong as the edge label, prefixed with “eRef_”. This allows for efficient identification during the mapping process and eliminates the possibility of unintentional name clashes on edges. A similar pattern is applied for attribute values. The left EObject vertex has a property prefixed with “eAttr_”, followed by the unique identifier of the “name” EAttribute. Again, this schema prevents name clashes and allows for fast identification of the corresponding meta-element. By following this schema, we can efficiently and unambiguously map each EMF model into its graph representation, and vice versa.

There are several additional details to be considered which are not represented in this figure. For example, Ecore allows to define an EReference which is many-valued and has a fixed ordering for its targets. By definition, edges on a vertex are unordered. Hence, we need to assign explicit numeric “order” attributes to these edges to retain this information. Even though such corner cases do exist and require special attention during the design of the mapping algorithm, the overall graph format is very concise, especially when compared to the large number of tables required in equivalent SQL representations.

### EQuery

Storing and retrieving models are basic capabilities that are offered by all model repositories. However, only very few tools allow for queries that operate on model content, such as CDO. Often, languages like *OCL* [53], *EOL* [42] or the *Hibernate Query Language* (HQL^20^) are employed for this purpose. HQL translates directly into SQL queries, utilizing the object-relational mapping information. From the modeling perspective, HQL is therefore a rather low-level language that is furthermore bound specifically to stores implemented in SQL. It operates on *storage level*, as opposed to working on the *model level*. OCL allows for model-level queries, but the execution of these statements often suffers from poor performance on larger instance models. Both OCL and HQL require that their statements are written as *plain strings* in an application, effectively circumventing any validation by compilers.

In the context of ChronoSphere, we introduce a new query language called *EQuery*. It uses familiar syntax and is implemented as an internal domain-specific language (DSL) embedded in Java itself. EQuery is based on *traversals* rather than declarative statements. Queries form an integral part of an application’s business logic, and embedding them directly into the Java source code has many benefits. Application developers can make full use of the Java compiler for validation, and benefit from their Java IDEs when it comes to editor support features, such as code completion and syntax highlighting. Queries will never go out of sync with the code that operates on them, and Java-level type safety is also preserved at compile time, which cannot be achieved with string-based query formats. Finally, EQuery also defines generic traversal steps that accept Java *Lambda Expressions*, which greatly enhances the flexibility and expressivity of queries.

EQuery works by first stating a *starting element*, then *navigating* the model from there to arrive at the desired *result element(s)*. In between, EQuery allows for a wide range of operations, including filters, loops and subqueries. Before execution, the traversals are scanned and optimized to make best use of the indices maintained by ChronoSphere [R5]. Internally, those queries are translated to graph traversal queries on the underlying *ChronoGraph* structure. Those queries allow for highly efficient, stream-based lazy evaluation. The entire EQuery framework is also easily extensible with new operations, such as the evaluation of OCL statements [53] or expressions in the Epsilon Object Language [42]. There is also an ongoing research project for translating OCL statements directly into graph traversals called Mogwaï [11], which might also be incorporated into our framework in the future.

The remainder of this section is dedicated to examples on how to use EQuery. We use the IT Landscape metamodel for this purpose, as shown in Fig. 20.
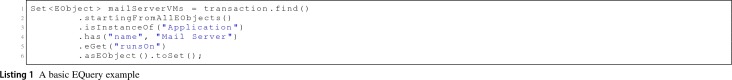


Listing 1 shows a basic example of the EQuery syntax. Here, we start our traversal from the set of all EObjects in our model. We then narrow the search space by restricting the elements to have an EClass named “Application”. We then filter the EAttributes of the EObjects and look for Applications with a “name” equal to “Mail Server”. Then, we *navigate* along the “runsOn” EReference using the familiar eGet(...) syntax. The result is a stream of arbitrary objects^21^ which needs to be filtered to include only EObjects. Finally, we convert the stream into a Set for further processing. All of this is expressed within regular Java code.
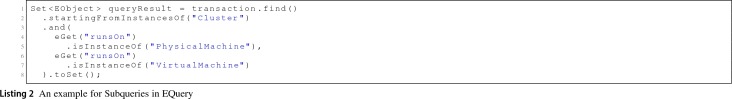


Listing 2 shows a query that retrieves all clusters which are mixed, i.e., run on physical as well as virtualized hardware. The query starts from the set of all clusters and then specifies an and(...) step. This step will filter out all EObjects where at least one of the provided subqueries does not produce any object. Each subquery starts at the same object, which is the one which was passed into the and step. In our example, we pass in a cluster object into the and step, and check if it runs on at least one physical and at least one virtual machine.
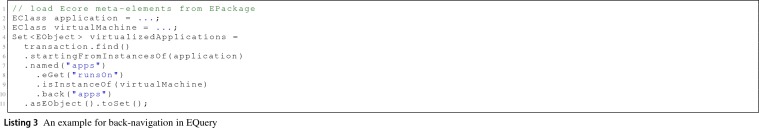


Our final query example in Listing 3 involves the retrieval of all Applications which run on virtualized hardware. To do so, we start from all applications in our model, and create a *name* for this traversal step (“apps”). This name can be arbitrary and is not connected to the model in any way; it merely acts as a *marker* in our query. From the applications, we navigate along the “runsOn” EReference to the virtual machines. For every application where this navigation step produced a valid result (i.e., application.eGet("runsOn") is non-empty), we check if at least one of the resulting EObjects is of type Virtual Machine. If there is such an instance, we navigate back to the previously marked “apps” position. This produces a stream that only contains EObjects that have at least one target for the “runsOn” EReference which is a Virtual Machine. It is important to note here that the back step will only be performed if at least one element passed the previous step. This style of backtracking works in arbitrary distances between query steps, and the programmer can define an arbitrary number of (uniquely) named steps to jump back to. They are useful in cases where the required filter condition is not on an element itself but on its neighbors, or in order to avoid long backtracking navigation. A final detail worth mentioning in Listing 3 is that we first retrieve the EClasses from the EPackage. In most places, the EQuery API allows the programmer to choose between passing a name (EClass name, EAttribute name...) or a variable of the appropriate type. Generally speaking, passing in the metamodel element directly is more efficient than specifying it by name. In all further listings, we will not include the variable definitions.

#### EQuery validation and type safety

As EQuery statements are fragments of Java code, they are subject to the Java type system and will automatically be checked by the Java compiler. Furthermore, as there is no media disruption between the query and the code which surrounds it, the compiler will also assert that the result of the query is of the type expected by the surrounding code. For example, in Listing 3 the compiler can check that the result is of type ${\texttt {Set<EObject>}}$. Aside from type errors, the compiler will also check that the general syntactic structure of the query is correct (e.g., no unbalanced braces, no mistyped operators...). Among the errors which will not be noticed by the Java compiler are mistyped names of metamodel elements and their types. For example, eGet(“name”) is always correct according to the Java compiler, even if the EAttribute name does not exist in the current metamodel. Furthermore, the Java compiler cannot infer that eGet(“name”) will produce an element of type String; all it knows is that it produces some result Object. For such cases, EQuery provides content filtering and casting methods (e.g., asEObject(), asNumber()...) which first apply an instanceof filter and then downcast the passing elements.

OCL takes a very different approach. From the perspective of a Java developer, OCL is an *external* DSL, which means that an OCL expression is embedded into a Java program as a string literal. By using tools such as the Dresden OCL compiler [12], it is possible to type-check an OCL statement, provided that both the statement and the corresponding metamodel are known. However, even though this is a strong validation, it cannot check the interactions between the query literal and the application which executes the query. On a Java API level, the result of an ocl.evaluate(literal) method call will always be of type Object which then needs to be downcast to the expected type. As both the content of the OCL string literal as well as the downcast itself can be changed freely without causing type errors from the Java compiler, we argue that this method does not provide full type safety for application developers. This is not limited to OCL: all query languages which rely on string literal representation (such as SQL and HQL) also suffer from the same issue.

Due to the fact that in our use case the metamodel is evolving dynamically at runtime and queries have to be created dynamically rather than coming from design-time-constant string literals, we decided to implement our query language as a Java-embedded DSL to retain as much type safety as possible by relying on the Java type system.

### Metamodel evolution

One of the major benefits of employing model repositories is the freedom of defining a custom, domain-specific metamodel for any given use case. In practice, users often cannot take full advantage of this benefit because they are hampered by the lack of proper tool support, in particular in cases where the metamodel evolves over the duration of a project [37]. These cases are very common in industrial contexts with long-running endeavors. In traditional enterprise software engineering scenarios, developers create database scripts that migrate the database schema (and contained data) from one version to the next. There is a wide variety of tools for this purpose (e.g., Flyway^22^ and LiquiBase^23^). In a model-based environment, this translates to the concept of *metamodel evolution* [R3], sometimes also referred to as *metamodel adaptation* [74]. The key challenge of metamodel evolution is to keep the instance model consistent with the metamodel, i.e., the instances need to be *co-adapted* such that they conform to the new metamodel [9].

**Fig. 18:**
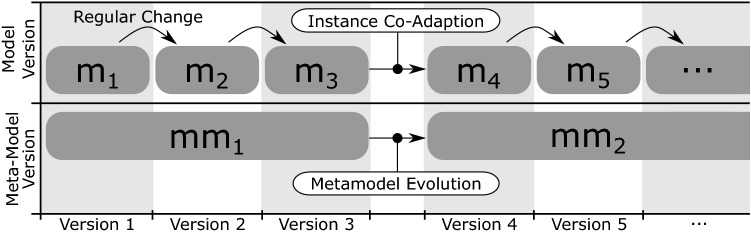
Metamodel evolution in ChronoSphere [28]

For some evolutionary metamodel changes, no instance co-adaptation is required. For example, when adding a new EAttribute to an existing EClass, the existing instances are still valid (provided that the attribute is not mandatory), they just have no value set for the new attribute. Other basic examples include the addition of new EClasses or increasing the multiplicity of an EAttribute from multiplicity-one to multiplicity-many. However, far more complex examples exist as well, and in many cases, fully automatic and deterministic instance co-adaptation is not possible. Cicchetti et al. refer to such cases as *unresolvable breaking changes* [9]. For instance, we consider a metamodel that contains an EClass*A*. The next version of the same metamodel does not contain *A* anymore, but a new EClass named *B* instead. Even though there are algorithms for model differencing [16, 40, 72], in the absence of unique identifiers (e.g., UUIDs) and change logs we cannot tell if *A* was removed and *B* was added, or if *A* was merely renamed to *B*. In the first case, we would have to delete all instances of *A*, in the second case we would need to migrate them to become instances of *B*. This basic example shows that instance co-adaptation requires *semantic information* about the change, which is only available to the *application developer*. For this reason, ChronoSphere provides an API for managing metamodel evolution with instance co-adaptation [R3]. Rose et al. provide a summary of related approaches [60]. This in-place transformation approach is in line with Wimmer [75] and Meyers [48], with the notable difference that we propose a Java API instead of ATL processes or DSLs [35, 59]. The concept is also similar to the Model Change Language [49]. This API offers three different modes of operation:

- *Changes without need for instance adaptation*
  This kind of evolution is intended for the most basic changes that do not require any kind of instance co-adaptation. Examples include adding EClasses, adding EAttributes, or increasing feature multiplicities from one to many. This category is also known as *non-breaking changes* [9]. The developer only provides the new version of the metamodel and loads it into ChronoSphere, which will create a new version in the history.
- *One-to-one correspondence*
  When instance co-adaptation is required, a common case is that each EObject from the old model will still correspond to (at most) one EObject in the new model. Examples for changes in this category include the renaming of EClasses and EAttributes. For such cases, the ChronoSphere metamodel evolution engine provides the developer with a predefined evolution process and a predefined element iteration order. The developer implements an *Incubator* that is responsible for either migrating a given EObject to match the new metamodel, or deleting it if it is obsolete. The Incubator is specific to a given source and target metamodel and contains the semantics and domain-specific constraints of the migration, expressed in Java source code.
- *Generic adaptation*
  In more complex cases, a one-to-one correspondence of elements can no longer be established, for example when an EClass is refactored and split up into two separate classes. In such cases, ChronoSphere provides a generic *Evolution Controller* interface that is in full control over the instance co-adaptation. It receives the *migration context*, which provides utility methods for querying the old and new model states. The migration process as well as the iteration order of elements are defined by the implementation of the controller. For that reason, implementing an evolution controller is the most powerful and expressive way of defining a migration, but also the most technically challenging one that entails the highest effort for the developer. Just like the incubators from the one-to-one correspondence case, such migration controllers are specific to a given source and target metamodel version.

By offering these features, we implement the metamodel evolution requirement [R3]. Since we only adapt the latest version of the model to the new metamodel, the old model instances still conform to their corresponding metamodel. We must not touch these instances, because this would violate the requirements for versioning and traceability of changes [R8, R9]. Hence, we need to put the metamodel under version control as well.

As shown in Fig. 18, every version in every branch of the model can have its own metamodel to which it corresponds. A direct consequence of this approach is that the application developer needs to be aware of those (potentially) multiple metamodels, and create queries dynamically based on that metamodel. While this will entail additional efforts in development, it is the only fully consistent way of managing versioned models with evolving metamodels.

The alternative would be to retroactively adapt every stored version of the model to a single new metamodel. However, since this adaptation process is not guaranteed to conserve all information (e.g., consider a new metamodel where an EAttribute has been deleted), we would not be able to guarantee traceability anymore. Consequently, we would introduce a considerable threat to the validity of audits. By storing a new metamodel alongside the co-adapted instance model, we restrict the impact of a metamodel evolution to a single version (e.g., the version that simultaneously introduces $m_{4}$ and ${mm_{2}}$ in Fig. 18) and can still guarantee traceability in the remaining sections of our data. As we will discuss in the remainder of this section, in our approach, we can guarantee traceability even across metamodel evolutions.
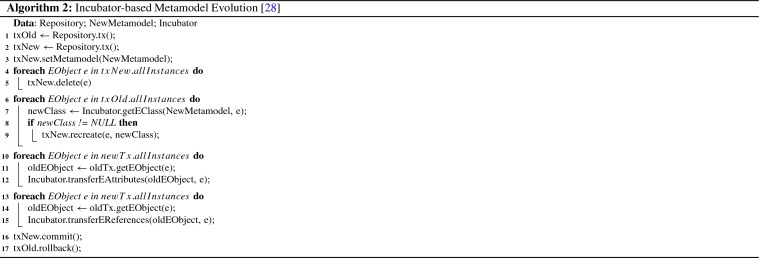


Algorithm 2 shows how metamodel evolution works in ChronoSphere when using an Incubator. In the beginning of the metamodel evolution algorithm, we open *two* transactions on the repository, and we refer to them as *txOld* and *txNew*. We will use *txOld* in order to read the repository state before the evolution has occurred, and *txNew* to perform our modifications. We assume that *txOld* contains a metamodel and a corresponding instance model (otherwise the evolution is a regular insertion). It is crucial at this point that these two transactions are able to work in parallel, and are furthermore completely *isolated* from each other. Our first actual modification is to *override* the previous metamodel in txNew. We can safely do so because the original is still stored in txOld. There is no metamodel differencing taking place in this phase, we perform a plain overwrite. We initially delete all elements in txNew (lines 4 to 5) and start with an empty instance model. Afterward, we begin our first instance evolution phase (lines 6 to 9). We iterate over all EObjects stored in the old repository state, and ask our Incubator for a new EClass for this particular EObject. If there is a corresponding EClass in the new metamodel, we *recreate* the EObject with the same ID, preserving the historical traceability link. Otherwise, we discard the EObject. In lines 10 through 12, we iterate over the elements that received a new EClass previously and look for their counterparts in txOld. We ask the Incubator to transfer any desired EAttribute values from the old version to the new one, which may also involve a value transformation step. For the fulfillment of all of its tasks, the Incubator has access to the Ecore API as well as the ChronoSphere API, allowing for very clean and expressive implementations. Finally, we construct the EReference instances by iterating over the EObjects again (lines 13 to 15). Once more, the Incubator is responsible for the actual semantics. In the last phase, we perform the commit that persists our changes to disk (and creates a new entry in the version history), and roll back the historical transaction.

Overall, we have maintained our traceability links (by retaining the IDs of EObjects) and performed a metamodel evolution with instance adaptation that is ACID safe and creates a clean history without partially evolved intermediate states. The evolution process with a full-fledged Evolution Controller works in much the same way. The primary difference is that the lines 6 through 15 are replaced by a call to the controller, allowing for a maximum of flexibility in the controller implementation. This algorithm requires efficient management of RAM in practice, in particular when working with larger models. Since *txNew* needs to manage all changes applied to all model elements, the change set can grow to very large sizes. By utilizing incremental commits, we can mitigate the problem by flushing batches to disk while providing the same level of ACID safety and equivalent histories.

### Advantages and limitations of the incubator approach

Using the incubator algorithm as described in section reduces the amount of manual coding required to perform a metamodel evolution with instance adaptation. The incubator assists the programmer in the common case that an EObject before the metamodel evolution conforms to at most one EObject after the metamodel evolution. This covers a lot of cases, including:

- Removal of classes
- Addition of new classes
- Renaming of any metamodel element
- Any changes to EAttributes and EReferences
- Any combination of changes listed above

In all of these cases, the incubator provides utilities such as a fixed migration process and a fixed element iteration order. The goal of the incubator approach is to drastically reduce the required amount of manual coding for common cases. However, it is not applicable in all scenarios. For example, when a single class *C* is split into two classes ($C_{1}$ and $C_{2}$), then each EObject *E* conforming to *C* must be split into two EObjects $E_{1}$ and $E_{2}$, where $E_{1}$ becomes an instance of $C_{1}$, and $E_{2}$ an instance of $C_{2}$. Cases such as this (which are less common and often fairly complex) are not covered by the incubator. In such cases, the programmer needs to implement the migration code manually. We consider the current features to be a baseline that provides the necessary raw functionality. We aim to build more sophisticated and user-friendly evolution mechanisms on top in the future, gradually reducing the amount of required manual coding (see Sect. 10).

**Fig. 19:**
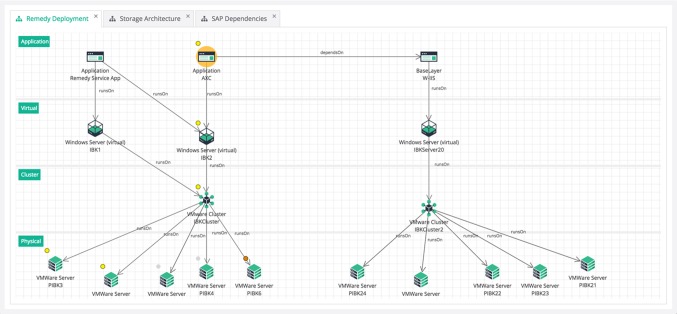
Analyzing transitive dependencies of IT assets in Txture’s interactive visualizations

### Transaction and versioning concepts

Transactional safety [R6] is the foundation of all features in ChronoSphere which are related to collaboration and evolution. Originally coined by the database community, this concept has since been adopted by other domains as well. In the context of modeling and model repositories, transactional safety implies that several clients can work in parallel on a model without interfering with each other.

In ChronoSphere, a *client*^24^ requests a transaction, then operates on it by executing queries and applying changes locally in this transaction. Afterward, the transaction can either be *committed* (local changes will be made available globally by creating a new version) or *rolled back* (reverting all local changes). The *isolation* property defined in the SQL Standard [38] states that any operation executed within a transaction *must not be affected* by other, concurrent transactions [R6]. In order to achieve the highest possible isolation level (*serializable* [38], also known as *snapshot isolation*), databases traditionally either need to perform excessive pessimistic locking, or allocate considerable amounts of RAM to open transactions in order to retain duplicates of concurrently modified entries. Thanks to its versioning capabilities, ChronoSphere can provide snapshot isolation with minimal locking, no additional memory overhead and without sacrificing performance. This is a direct consequence of our design: once a model version is committed, it is effectively immutable. Further changes will create new versions. Therefore, as long as a client is working on any given version (i.e., the used transaction timestamp does not change), the model content will not change, thus guaranteeing snapshot isolation.

## Industrial case study

At the time of writing this document, ChronoSphere and its sub-components are already being used in production in industry. They are embedded in the commercial IT Landscape documentation tool *Txture*,^25^ which is in turn deployed on-site at several customers. In this section, we explore how the interplay between Txture and ChronoSphere works and how ChronoSphere contributes to the use cases of Txture.

**Fig. 20:**
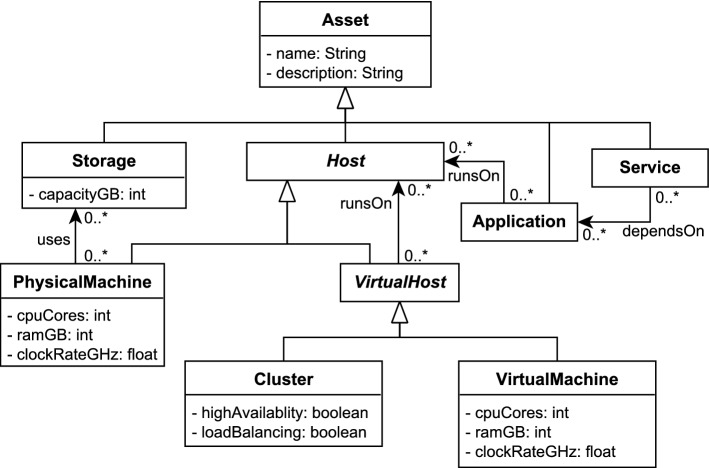
Metamodel for IT landscapes (simplified)

Txture is a software tool for documenting the IT Landscape of companies. The customers of Txture employ it for several different use cases, including impact analysis, history analysis, enterprise architecture management, transformation planning and as a supportive tool for datacenter outsourcing. Txture uses ChronoSphere as its primary data storage. Its clients interact with Txture via a web-based user interface. This UI offers interactive, query-based near-real-time visualizations to the end user. The supported visualization types include graph-based views, tables and charts. The graph-based views (as shown in Fig. 19) are suitable for many IT Operation and Enterprise Architecture use cases, because they allow the user to navigate the connections between model elements in an interactive way, expanding areas of interest and collapsing others into groups (or hiding them entirely). This workflow allows to abstract away from distracting details on the fly. Each navigation step performed by the user on the front-end is translated into a query step and added to the existing base query. The server core is responsible for executing these queries and directly relies upon the capabilities of ChronoSphere. The metamodel in Txture can be adapted to suit the needs of the customer (usually starting from a best-practice model synthesized from past projects; a simplified version is shown in Fig. 20). Therefore, queries cannot be hard-coded; they need to be created on the fly in order to conform to the metamodel. In a string-based approach (e.g., OCL strings), the only solution would involve building queries from individual pieces via string concatenations, which is an error-prone process. In this approach, the Java compiler cannot offer any checks with respect to the content of a query string. EQuery is implemented as a Java-embedded internal DSL (c.f. Sect. 6.2) which is beneficial for this scenario, as queries can be assembled dynamically one step at a time without the need for string concatenations. As queries are expressed as Java code and ChronoSphere is intended as a tool for application developers, further measures are required to make ad-hoc queries available to end-users. Txture implements a custom query syntax for end-users which is then translated into EQuery statements. Another approach for ad-hoc queries could be to employ a JVM scripting language such as Groovy,^26^ which would allow end-users to type their EQuery statements directly into a text field and evaluate them.

The incremental commits offered by ChronoSphere (see Sect. 4.2) are also used by Txture. Data imports from external data sources, most notably from a *Configuration Management Database* (CMDB [7]), often produce a large quantity of new elements to insert into the model. Incremental commits allow to perform this insertion in a number of smaller successive steps, avoiding memory limitation issues on the server while preserving a history that does not contain any intermediate (incomplete) states. Furthermore, this preserves the atomic nature of imports: even if previous incremental commit portions have already been written to disk, an error that occurs toward the end of the process can still cancel the entire process without corrupting the database content.

A feature that is used in particular by Enterprise Architects in Txture is the planning feature. This allows an architect to apply arbitrary changes onto the current state of the model, without making them part of the “as-is” architecture or making them visible to anybody else. Txture provides the same suite of analysis tools for the resulting plans that is also available on the “as-is” model, in addition to comparison features that allow to analyze the impact of the plan on the real model. This is realized using the branching feature of ChronoSphere. When a new plan is created by a user, Txture switches the currently selected branch for that user from the master (“as-is”) branch to a new one. Since this branching is lightweight in ChronoSphere, the user does not experience any waiting time during this switch even when creating new plans on top of large models.

A similar argument can be made for the Time Machine feature in Txture. By selecting a date and time in the past, a user can move the entire client to that particular point in time and review the contents of all existing visualizations calculated on that model state. The versioning feature of ChronoSphere acts as the enabling technology here: Txture uses the date and time selected by the user on the user interface as the request timestamp for the ChronoSphere transaction. As all queries are timestamp-agnostic, all visualizations adapt to the new situation automatically as their backing queries are being re-evaluated by the server.

The fact that each revision is immutable in ChronoSphere also brings additional advantages that are utilized by Txture. Any client starts out on the latest revision of the “as-is” model at the time of logging in. When a new model version is created at the server, a notification is sent out to all clients, informing them about the existence of this new update. On most systems, this would entail that the client has to react immediately and fetch the new information from the server in order to prevent synchronization issues. Txture clients are not forced to interrupt their current task, because the version a client is currently viewing is immutable. The user can continue to analyze the currently selected model version indefinitely as the server will yield consistently reproducible query results for any version. Should the user choose to switch to the latest version, the transaction timestamp associated with the session is updated to the most recent one, refreshing all query results and client views in the process.

## Performance evaluation

In comparison with other repository solutions, ChronoSphere offers several additional features while making fewer assumptions about the input model (e.g., we do not assume the metamodel to remain constant over time). This rises the question how well our approach can perform in comparison with other solutions. In this section, we present a comparative benchmark between ChronoSphere and the Eclipse CDO repository. We specifically selected CDO for three reasons:

- CDO is widely considered to be the *“gold standard”* in model repositories.
- In the spectrum of competing solutions, CDO is closest to ChronoSphere with respect to features.
- CDO is based on a relational store, which allows for a discussion of individual advantages and drawbacks when comparing it to our graph-based approach.

For this benchmark, we operate on a fixed Ecore model (as required by CDO). This model uses the Metamodel for IT Landscape Management (Fig. 20 shows a simplified version, the full Ecore file is available online^27^). Since instance models in the IT Landscape environment are sensitive information and therefore confidential, we created a synthetic model in collaboration with our industry partners at Txture. This model exhibits the same inner structure (element counts, associations, ...) as a typical real-world model. The instance model file is available online.^28^ It consists of approximately 200000 EObjects.

This benchmark includes four individual scenarios. The scenarios have been synthesized from the most commonly used queries in Txture, as introduced in Sect. 1. In each scenario, we grant 5GB of RAM to the Java Virtual Machine (Java 1.8 Update 161) which executes the code. It is possible to run the benchmark with less RAM, however in such cases, the garbage collector can impact the individual runtimes in unpredictable ways. The relevant hardware in our test system included an Intel i7 5820K CPU (3.30GHz) and a Crucial CT500MX SSD, operated by Windows 10. All tests were executed on a pre-warmed JVM. Each figure in the benchmark shows the results of the scenario, averaged over 10 independent executions. Variances are not included in the figures due to their small magnitudes. We would like to emphasize that CDO offers several ways of executing queries. One of them is the usage of the Hibernate Query Language (HQL^29^). This language requires deep knowledge of the underlying storage, as the user needs to be aware of the way in which database tables are generated from model classes. Another way of querying the data in CDO is by using its programming interface directly. The third and final way for querying data in CDO is by specifying OCL [53] queries. We argue that OCL queries are the only option to formulate queries on the abstraction level of the model, without requiring any knowledge of the underlying storage. In this benchmark, we will therefore only utilize OCL queries in CDO. We use the latest stable version of CDO as of January 2018.

### Model insertion

The first benchmark scenario consists of loading the 200000 EObjects into the system and persisting them to disk.

**Fig. 21:**
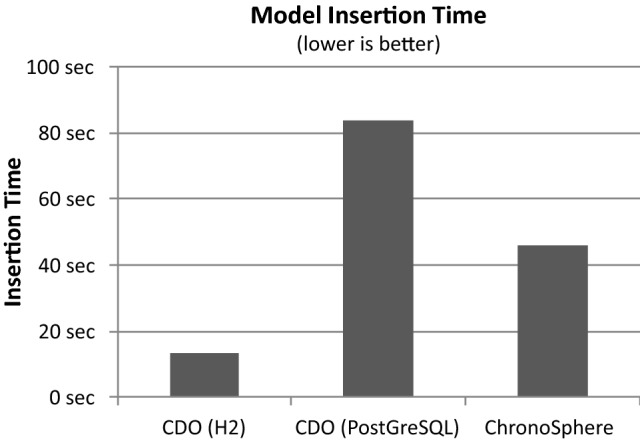
Insertion performance

As Fig. 21 shows, there are significant performance differences during model insertion when using different database back-ends for CDO. The embedded H2 database offers faster insertion speed compared to PostGreSQL. ChronoSphere is the middle ground between the two. The primary factor which is slowing ChronoSphere down in this comparison is the fact that both H2 and PostGreSQL have access to fixed schemas, which allows for a very compact binary representation of each EObject. ChronoSphere uses a more flexible approach, resulting in less densely packed data structures and consecutively lower insertion speed. CDO accomplishes this performance also by deliberately working without foreign key constraints in the database itself, thus forfeiting one of the biggest advantages of relational storage systems.

### Assets By Name

A very common task for any repository is to allow a user to find an element by its name. In this scenario, we are searching for a Physical Machine by name. We use the following queries:
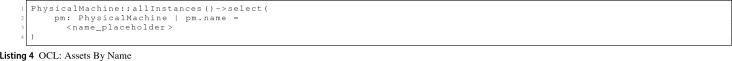

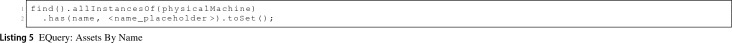


**Fig. 22:**
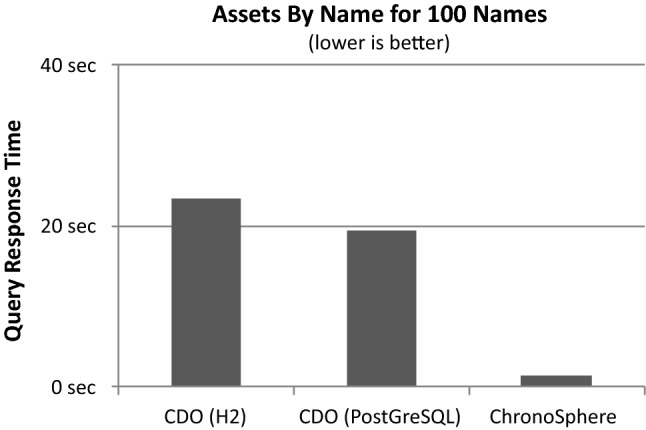
Assets By Name performance

We repeat each query 100 times, using a different replacement for the ${\texttt {<name\_placeholder>}}$ in every iteration. Using the same name in every execution would entail the possibility that the tool under test is simply returning a cached query result which contradicts our measurement goals (Fig. 22).

Given the fact that relational databases have been designed especially for this kind of query, it might lead to the assumption that CDO is capable of answering this query very efficiently. However, it turns out that the CDO query evaluation engine for OCL does not inspect the provided OCL statement for optimization possibilities. Instead of creating a suitable SQL statement and forwarding it to the underlying database, the OCL statement is evaluated simply by iterating over the EObjects in memory. Another shortcoming of CDO is the fact that it does not create secondary indices on model data, and also does not offer such an option through its API. While it is possible to manually create secondary indices in the underlying database, they will not be utilized by CDO. ChronoSphere analyzes and optimizes the passed query and utilizes a secondary index on the element name, allowing for greatly reduced query response times in this scenario.

### Root cause analysis

After retrieving a particular element by name, a common task in the IT Landscape context is to perform a *Root Cause Analysis*. Such an analysis attempts to track down the root cause for a failing asset. For example, an application might fail to operate properly because the underlying virtual machine fails. The query therefore needs to retrieve the transitive dependencies of a given asset. For this particular scenario, we consider the transitive dependencies of a Service asset down to the PhysicalMachine level. This is commonly referred to as the *deployment stack*. We measure the accumulated time for 1000 executions of this query on different initial start objects. The queries in OCL and EQuery are formulated as follows:
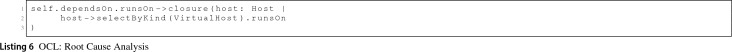

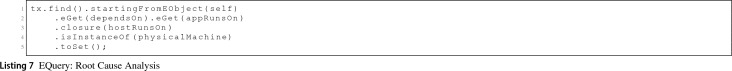


In this benchmark, we utilize the closure statement in both languages to navigate the transitive paths. We require this statement because the path length is unknown—a Virtual Machine may run on a Cluster which in turn is deployed on Virtual Machines. Such a query is very difficult to express in SQL and would require recursive JOIN operations. However, since CDO resolves OCL queries via in-memory iteration, it circumvents this issue.

**Fig. 23:**
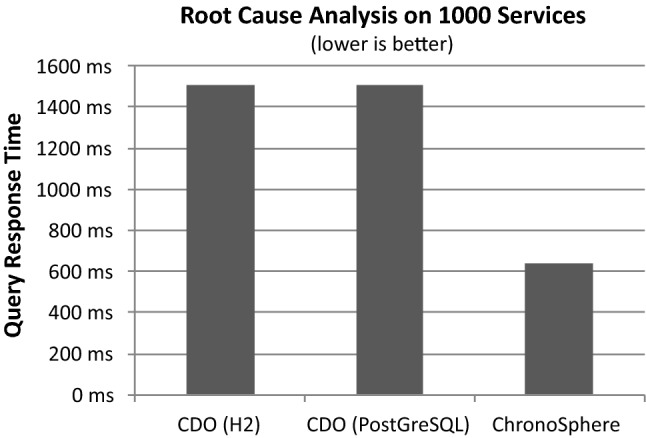
Root cause analysis performance

Figure 23 shows that CDO provides acceptable performance in this scenario; however, ChronoSphere outperforms it by a factor of 2. The reason for this speedup lies within the architecture of ChronoSphere: after consolidating the EQuery steps, it transforms them into a Gremlin graph traversal. This highly optimized traversal engine is then executing the query on the data structures provided by ChronoGraph. We avoid the overhead of the reflective EObject API completely, granting ChronoSphere faster query execution.

### Impact analysis

Root Cause Analysis, as introduced in the previous section, finds the cause of failure of a particular asset. The inverse question is also relevant in IT Landscape management: given an asset, we need to determine which higher-level assets are affected in case that this asset fails. For our model-level query, this means that we need to find the *incoming* transitive dependencies of a model element. For our benchmark, we perform the impact analysis from a Physical Machine all the way up to the Services. We formulated the corresponding queries as follows:
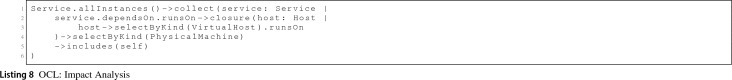

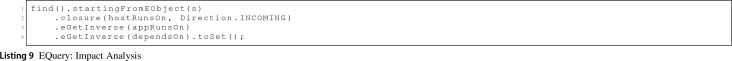


In this particular scenario, we encounter a shortcoming of OCL: it does not allow to formulate query steps across the incoming references of an object. This issue has been perceived by tool authors and there are extensions to OCL which allow for an *eInverse* step, e.g., in the Acceleo framework.^30^ However, as CDO only supports the official OCL standard, our options for formulating this query (without having to modify our metamodel) are very limited. We iterate over all services, build the deployment stack for each service, and then check if our Physical Machine in question is contained in the resulting set of elements.

**Fig. 24:**
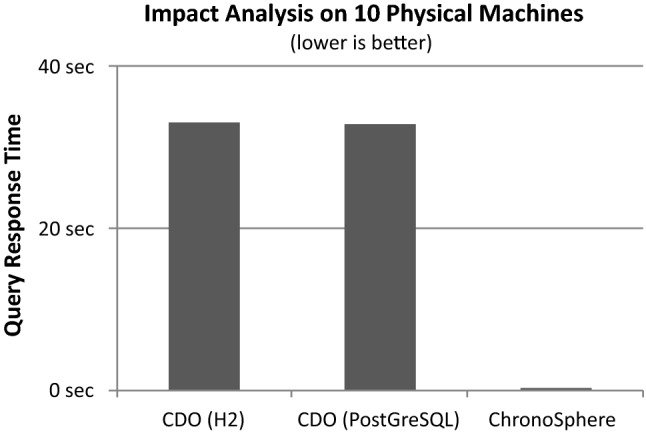
Impact analysis performance

As Fig. 24 clearly shows, the limitations of OCL severely impact the performance of the query in CDO. In contrast, the EQuery expression in ChronoSphere has approximately the same performance as the forward-navigating Root Cause Analysis query. Please note that we reduced the number of input elements from 1000 to 10 in this scenario in order to get results from CDO within a reasonable time frame. Multiplying the result of ChronoSphere by 100 yields the same results as in the Root Cause Analysis scenario. This demonstrates that ChronoSphere queries can navigate along outgoing and incoming references without any performance penalty.

### Threats to validity

We tried to achieve a comparison in this chapter which is as fair as possible. Nevertheless, some threats to the validity of our results could not be eliminated during the process. First of all, the exact behavior of the Just-in-Time Compiler of the Java Platform, as well as its garbage collector, cannot be completely pre-determined. This adds some inherent variance to the results, which we tried to mitigate by pre-warming the JVM and assigning sufficient RAM to the Java process. In the CDO cases, we implemented the queries on a CDO client, with the CDO Server process running on the same machine in order to eliminate network latency. CDO does offer an embedded mode; however, in our tests we unfortunately found this mode to be unstable and prone to a number of unpredictable runtime exceptions. One might argue that implementing the queries in HQL would have yielded better performance in CDO; however, we chose OCL because it operates on the model level rather than on the persistence level. Also, in HQL it is currently not possible to formulate queries which are recursive or rely on transitive closures, which we require in our benchmark scenarios. The employed model, even though crafted in collaboration with the experts at Txture to ensure real-life conditions, is a synthetic construct, which might lead to different results in real applications.

**Table 7:** Benchmark result summary (execution times in ms)

	CDO (H2)	CDO (PGSQL)	ChronoSphere
Model Loading	**13,404**.**5**	83,915.8	46,077.0
Root Cause Analysis	1509.6	1504.2	**636**.**3**
Impact Analysis	33,155.0	32,907.9	**12**.**5**
Assets By Name	23,292.3	19,470.0	**1517**.**4**

Bold value represents the best score in the respective row

### Benchmark summary

In this comparative evaluation (which is summarized in Table 7), we demonstrated that ChronoSphere offers competitive performance, even though it does not require a metamodel to be constant over time. The underlying graph database allows for schema-free model storage without sacrificing query execution speed. This benchmark also demonstrates that avoiding O/R-mapping techniques has a positive influence on the overall performance. We also showcased the expressiveness of our query framework, which allows for more flexible navigation than OCL. This advantage is crucial in the IT Landscape domain and generally beneficial in model analysis. Our results are also in line with the extensive comparative benchmark by Barmpis et. al [1] which demonstrates the advantages of graph-based storage over relational solutions for model persistence.

Please note that we focused exclusively on ChronoSphere in this evaluation. For an evaluation on ChronoDB [25] and ChronoGraph [27], we refer the interested reader to the respective publications.

## Discussion and related work

Over the years, a considerable number of model repositories have been developed by various authors. Pierantonio et. al provide a good overview in their paper [13]. In this section, we will compare our approach to other solutions which are conceptionally close to our repository. As our approach also entailed the development of lower-level infrastructure, we will also consider related work in those areas. This section is structured in a bottom-up fashion, starting with the related work in the area of versioned key-value stores and concluding with related work in the model repository area.

### Related Key-Value-Store versioning solutions

Database content versioning is a well-known topic. Early work in this area dates back to the 1986 when Richard Snodgrass published his article on *Temporal Databases* [69]. Adding a time dimension to the data stored in a database considerably increases the complexity of the data management, because the additional dimension introduces new demands regarding data consistency and several tried-and-true solutions are no longer applicable to the same extent as with non-versioned data (e.g., hash tables for caching). Key-value stores have become attractive formats for versioned data due to their simple nature when compared to relations, graphs or documents.

Sridhar Ramaswamy published a paper in 1997 on indexing for temporal databases [57]. He proposes an approach based on *validity ranges* for each entry to which he refers as *windows*. Each entry is valid from its insertion until its validity is explicitly terminated by an update or deletion. This transforms the problem of a search in a versioned database into an instance of the well-known *interval stabbing problem* [65]: given a set of intervals and a number, find all intervals containing this number. This approach strongly inspired our efforts. The major difference between the algorithms we employ in ChronoDB (c.f. Sect. 4.1) and Ramaswamy’s approach is that in our case, the upper limit of each validity window is given *implicitly* by the matrix structure. We therefore do not need to update a previously stored validity range in our database when a new version is added. This allows our data store to operate in a strictly *append-only* fashion, which entails a number of technical advantages in the implementation. Also, deletions of entries do not impact our data structure in any different way than inserts or modifications, which was an issue in Ramasway’s solution.

Felber et. al [21] propose a different solution. For every key, the store manages a *list* of values, each value corresponding to one version. This is a simple and effective system for managing elements with *independent* histories (e.g., wiki pages). However, this solution does not preserve the historical correlation between elements. For example, given a key $k_{1}$ with values $a_{1}$ and $a_{2}$, and a key $k_{2}$ with values $b_{1}$ and $b_{2}$, there is no way to tell if the entry $(k_{1},a_{1})$ existed at the same time as $(k_{1},b_{1})$, or $(k_{1},b_{2})$ or neither of them. This temporal correlation is crucial when individual values can contain references to one another, as it is the case with ChronoGraph.

Commercial database vendors also explored the possibilities for database content versioning. Lomet et.al [44, 45] developed an approach named *ImmortalDB* which was later integrated into Microsoft SQL Server. This solution is based on *history chains*: each entry refers to its predecessor via a pointer (alongside timestamp metadata). This approach allows for high performance of queries on the latest version of the data. However, as the request timestamps are moved further back in time, the query performance degrades linearly, as the history chains need to be traversed. ChronoDB avoids this problem and offers logarithmic access time to any entry, regardless of its age. Further commercial projects in this area include *Temporal Tables* in IBM DB2 [63] or Oracle’s *Flashback* technology [32]. The choice between history chains and time-indexing (as shown in Sect. 4) depends on the use case. History chains offer almost the same performance for queries on the latest version as an unversioned system, but when previous versions are requested, the performance decreases linearly with the age of the requested version, as all intermediate chain links need to be traversed. Time-indexed solutions are engineered to offer nearly identical query performance on any requested version, but the overall query performance decreases in a logarithmic fashion as new versions are being added. We mitigate this issue by splitting the data along the time axis, thus limiting the search space of each request (see Algorithm 1). For systems which primarily serve the latest version, history chains are a viable choice. However, in particular for the use case of IT Landscapes, the performance of historical queries matters to the end users, as the repository is used for audits and history analysis.

### Related graph versioning solutions

In comparison with Key-Value stores and SQL databases, graph databases as we know them today are a relatively new technology. Consequently, there are fewer approaches regarding content versioning.

Considering the large amount of development and quality assurance efforts that has been invested into existing graph databases (e.g., Neo4J or TitanDB), it is a tempting idea to integrate versioning in these systems rather than developing new ones. Castelltort and Laurent published one of the first papers [8] that seek to integrate versioning into general-purpose graph databases. This is achieved by creating a layout for a “meta-graph” that can contain and manage multiple versions of itself. The graph database contains this meta-graph, and incoming queries need to be aware of this structure in order to extract the information they require. As Castelltort and Laurent clearly show in their paper, the complexity of queries sharply increases in such a scenario. Due to the increased number of checks that need to be performed by each query, the performance inevitably degrades as well. Perhaps the largest drawback of this approach is that the *application* needs to be aware of and manage this additional layer of complexity. There are several different approaches for creating a layout for the meta-graph, e.g., the solution proposed by Taentzer et al. [71] which is based on modeling differences between versions as graphs. There is one central issue which is shared by all layouts: Given a suitable set of graph changes, they introduce vertices in the graph which have a very high degree of incoming and/or outgoing edges for the purpose of version control. Such *super vertices* represent a problematic corner case in any graph database and may lead to poor performance and storage space utilization. As ChronoGraph manages version control on a lower level, there is no need to introduce any additional graph elements in order to achieve versioning. The disadvantage of our solution in that regard is that a completely new implementation was required and existing graph databases could not be reused.

Other related approaches, e.g., by Semertzidis and Pitoura [66, 67] or by Han et al. [29], assume the existence of a series of graph snapshots as input to their solutions. These approaches do not aim for online transaction processing (OLTP) capabilities and focus on the analysis of a series of static graphs. A direct comparison with our approach is therefore not feasible. However, the data managed by ChronoGraph may serve as an input to those tools, as each graph revision can be extracted individually and consequently be treated as a series of snapshots.

### Related repository solutions

**Table 8:** Model repository feature comparison

Technology	F1	F2	F3	F4	F5	F6	F7	F8	F9	Deployment	Persistence
Eclipse CDO	✓	✓		✓	✓	✓		✓	✓	On Premise	SQL/Documents
MORSA	✓									On Premise	Documents
EMFStore	✓	✓		✓				✓		On Premise	XML Files
MagicDraw Teamwork Server	✓			✓				✓		On Premise	XML Files
MagicDraw Teamwork Cloud	✓	✓		✓	✓					On Premise	Key–Value Store
Hawk Model Indexer	✓				✓	✓				On Premise	Graph
Neo4EMF	✓				✓	✓				On Premise	Graph
GreyCat	✓	✓		✓	✓	✓				On Premise	Versioned Graph
**ChronoSphere**	✓	✓	✓	✓	✓	✓	✓	✓	✓	**On Premise**	**Versioned Graph**
GenMyModel	✓							✓		Cloud (SaaS)	SQL
MDEForge	✓		✓		✓	✓				Cloud (SaaS)	Documents

Table 8 shows a comparison of related model repositories based on the required features we established in Sect. 2. The table can be divided into two sections, which are *cloud (Software-as-a-Service)* solutions and *on premise* solutions. While cloud-based solutions for EAM models exist (e.g., Iteraplan^31^), more fine-grained IT Landscape models are widely considered to be very sensitive data in industry. Unauthorized access to such models could guide a potential attacker to the servers where the impact of the attack is maximized. Most companies therefore require an on premise deployment of the software that manages their IT Landscapes. This eliminates cloud-based tools such as *MDEForge* [3] and *GenMyModel* [14] from the list of possible repository candidates for IT Landscape models.

*Connected Data Objects (CDO)*^32^ is widely considered to be the gold standard of model repositories. This repository uses SQL databases to store model data in a versioned fashion. CDO handles the versioning process internally, it does not make use of versioning features in the underlying database. With respect to features, CDO is the most complete competitor to ChronoSphere. However, CDO exhibits several weaknesses when employed in practice [68]. In particular, the lack of any support for metamodel evolution motivated our decision to implement a novel model repository. ChronoSphere also avoids the Object-Relational Mapping (O/R-Mapping) process which is employed by CDO in order to transfer model data into and out of the underlying database. As we have shown in Sect. 6.1, there is a natural pattern for transforming model elements into graph elements (and vice versa), whereas O/R-Mapping is a fairly involved process [39], both conceptionally as well as with respect to resource usage (e.g., CPU and RAM). We also performed a comparative benchmark between CDO and ChronoSphere in Sect. 8.

Neo4EMF [4] was the first storage solution for EMF models that is based on graph database technology. This work has inspired and guided our efforts. ChronoSphere utilizes a similar model-to-graph mapping as Neo4EMF, albeit a different implementation for technical reasons. Neo4EMF showed the advantages of graph-based persistence, however it is a persistence framework rather than a model repository. Central features, such as versioning, branching and ACID transaction support, remain unaddressed by this technology.

Hawk Model Indexer [2] is another solution in the realm of model engineering that seeks to utilize graph-based persistence for models. As the name implies, Hawk is primarily an *indexer*. It is therefore not responsible for the actual model persistence, but rather for creating a secondary structure to speed up incoming queries. Hawk is intended to be used as an assistive technology and does not qualify as a standalone model repository. Just as with Neo4EMF, features like versioning and branching are not considered by this approach.

MORSA [54] was one of the first NoSQL model repositories. It stores models in a document-based backend (MongoDB^33^). The main features of MORSA include model versioning and persistence. However, in contrast to ChronoSphere, MORSA treats a model as a single atomic artifact. Queries on the model content are therefore not supported. Also, the versioning process takes place on the granularity of the entire model (rather than per-element as in ChronoSphere). MORSA is suitable for storing and retrieving hand-crafted models of smaller sizes. The large models generated by automated processes in IT Landscapes would introduce a prohibitive amount of overhead for whole-model versioning approaches.

EMFStore [41] is a model repository that operates on *model differences*, which are stored in files in an XML-based format. This allows EMFStore to support per-element versioning and branching as well as efficient storage utilization for long history chains. However, EMFStore does not offer support for model content indexing and/or querying. Retrieving a model version requires a *checkout* operation as seen in traditional version control systems, e.g., Git or SVN. The commercial tool *MagicDraw Teamwork Server*^34^ follows a similar approach as EMFStore. Teamwork Server internally stores the XMI [52] representation of a model in a folder controlled by SVN, which introduces similar scalability issues as discussed about MORSA. ChronoSphere follows a different approach: each version of each model element is accessible in logarithmic time without requiring a checkout procedure of the entire model. Also, ChronoShpere allows for indexing and querying of the model content in contrast to the other mentioned solutions in this category. Teamwork Server has been superseded by *MagicDraw Teamwork Cloud*^35^ which employs a per-element versioning approach and is based on Apache Cassandra. Even though this approach allows for a higher scalability, due to the nature of Cassandra, this solution cannot support ACID transactions. As of the current version (19.0), according to the official API documentation^36^ Teamwork Cloud does not offer any extended querying capabilities beyond retrieving a model as a whole and picking individual elements by ID. It does however utilize the same retrieval model as ChronoSphere, where elements are retrieved by stating their ID as well as their branch and timestamp.

An approach that is conceptually close to ChronoSphere, but does not declare itself as a model repository, is GreyCat^37^ [33, 34]. GreyCat stores models in versioned graphs. It is based on the Kevoree Modeling Framework [23] (KMF), which is an alternative to EMF that focuses on models-at-runtime scenarios. KMF metamodels can be automatically derived from Ecore definitions (hence we consider the metamodeling process to be Ecore compliant in Table 8). Much like ChronoGraph, GreyCat implements its own graph layer on top of a NoSQL storage. In its storage layer, GreyCat uses history chains (*traces* in GreyCat terminology) which consist of change operations. Thus, GreyCat utilizes difference-based versioning, whereas ChronoGraph employs state-based versioning. While GreyCat also implements a property graph, it does not support the TinkerPop API. A major difference that sets ChronoSphere and GreyCat apart is the fact that GreyCat is heavily relying on code generation. This entails that the metamodel for GreyCat is fixed for all versions and branches, and metamodel evolution cannot be supported in the same way as it is possible in ChronoSphere. GreyCat (and its predecessors) and ChronoSphere have been developed during the same time periods as independent projects, which is the reason why neither of them is built on top of the other. The existence of two independent solutions also highlights both the importance of versioned model storage as well as the suitability of property graphs for this task.

Further related work specifically in the IT Landscape domain includes Configuration Management Databases (CMDBs). There is a wide variety of commercial products on the market (e.g., BMC Atrium,^38^ ServiceNow^39^ or HP Universal CMDB^40^). A direct comparison with ChronoSphere is infeasible because CMDBs are tightly bound to their application domain, whereas our solution is generic and domain independent. The metamodel in a CMDB is usually fixed and tailored toward the IT operations use case. Versioning capabilities are also found in CMDB products, but they are often limited to the history of single elements (i.e., it is not possible to move an entire view with multiple elements back in time). Overall, we do not consider CMDBs to be model repositories because they do not utilize a metamodeling language (e.g., Ecore), they are domain-specific and operate on a fixed metamodel. However, a model repository can be used as the back-end of a CMDB or EAM application.

**Table 9:** The chronos technology stack

Technology	Classification	Source Code Repository
ChronoDB [25]	Versioned Key-Value Store	https://github.com/MartinHaeusler/chronos/tree/master/org.chronos.chronodb
ChronoGraph [27]	Versioned TinkerPop Graph Database	https://github.com/MartinHaeusler/chronos/tree/master/org.chronos.chronograph
ChronoSphere [28]	Ecore Model Repository	https://github.com/MartinHaeusler/chronos/tree/master/org.chronos.chronosphere

Table 8 shows two important facts. On the one hand all of the features we implemented in ChronoSphere (except for historical archiving) are present in at least one related tool. This emphasizes the importance of the chosen feature set. On the other hand, this table shows that ChronoSphere also fulfills all requirements of a *general-purpose* model repository and is therefore not restricted to IT Landscape modeling in any way.

### ChronoSphere as a generic model repository

ChronoSphere has been created specifically for the use case of IT Landscape documentation. However, the resulting concepts and software are generic and have no dependencies to this particular domain. As a general-purpose EMF model repository with a rich feature set, ChronoSphere can be applied in a wide range of use cases, in particular in models-at-runtime scenarios. In the context of this paper, we decided to focus on the domain for which the tool was originally created. While the features of ChronoSphere are generic, it is optimized for the workloads expected in the IT Landscape domain (model sizes, frequency of insertions and queries, number of concurrent users...). We will conduct further studies in the future where we apply ChronoSphere in different domains.

## Outlook and future work

ChronoSphere and its components currently operate exclusively in local deployments. However, as other projects (e.g., Neo4J and TitanDB) have shown, graph databases lend themselves well to distribution across several machines. One of our future goals is to create a distributed version of ChronoGraph for greater scalability. The fact that this database operates in a versioned, append-only fashion should ease this transition as the common problem of encountering stale data is eliminated by design. Due to the chosen layer separation, the code base of ChronoSphere itself will remain largely unchanged. Overall, we hope to achieve a distributed model repository that can scale well with even larger models and higher numbers of concurrent users.

EQuery, the query framework introduced by ChronoSphere, is constantly evolving. With inspiration from Project Mogwaï [11], we aim for the inclusion of OCL expression evaluation into our EQuery framework. This will allow programmers to have a ocl(String) step in the query where the OCL statement is provided as a string. This string will then be analyzed and transformed into a graph query, which is then used as a subquery in the overall evaluation. By similar means, we intend to integrate the Epsilon Object Language [42] as sub-expressions in our query framework. This will allow ChronoSphere to offer support for several different query languages within the same underlying engine.

The metamodel evolution facilities in ChronoSphere are intended as a baseline. They offer the atomic commands required by any evolution mechanism and focus on raw functionality. However, certain use cases may require more sophisticated approaches, e.g., transformations based on differences between two given metamodels. We plan on introducing additional abstraction layers on top of the current imperative design in order to support such transformations, gradually reducing and ultimately eliminating the required amount of manual coding efforts.

Finally, we will continue our ongoing efforts to increase the overall code quality, test coverage, documentation and performance of the implementation (Table 9). ChronoSphere is a work in progress project that uses a large amount of software that was developed specifically for this purpose and consequently includes less off-the-shelf software components than other projects. There is still a lot of room for improvement in the implementation details which we intend to explore in the near future.

## Summary

In this paper, we presented ChronoSphere, a novel open-source EMF model repository. This model repository was designed and implemented to support large IT Landscape models in industrial contexts, but is generic and can be employed in any EMF-based modeling scenario. We analyzed how we inferred the requirements from the IT Landscape context and how they relate to the technical features offered by ChronoSphere. We then focused on the concepts behind our repository implementation which also contributed to the state-of-the-art in versioned data storage and graph databases. We discussed the commonalities and differences of our solution with respect to related repository technology. Our concepts and technology were evaluated in a case study where ChronoSphere is used as the primary storage backend by the industrial IT Landscape modeling and analysis tool Txture. Building on top of the use cases of this case study we performed a comparative benchmark with a state-of-the-art model repository and demonstrated the competitive performance of our solution. ChronoSphere is a fresh impulse in the area of model repositories not only in terms of its features and implemented standards, but first and foremost in that it provides the entire data management stack, allowing for a clean and consistent architecture. As all of the individual components are available as open-source software, each aspect is accessible for experimentation and innovation in future research projects. ChronoSphere is an all-new approach to model repositories, and we hope that it will serve as a platform for future projects in research and industry alike.

## Footnotes

1 www.txture.io.

2 https://neo4j.com/.

3 http://titan.thinkaurelius.com/.

4 http://tinkerpop.apache.org/.

5 https://github.com/MartinHaeusler/chronos/tree/master/org.chronos.chronograph.

6 https://github.com/MartinHaeusler/chronos/tree/master/org.chronos.chronodb.

7 https://github.com/EsotericSoftware/kryo.

8 https://github.com/cojen/Tupl.

9 In most cases, linear search in main memory is still faster than performing $\mathcal {O}(\hbox {log}(n))$ disk accesses. Also, the cache usually does not contain all data, so these lists will remain short.

10 http://s3.thinkaurelius.com/docs/titan/1.0.0/schema.html.

11 https://orientdb.com/docs/last/Tutorial-Using-schema-with-graphs.html.

12 All circles in Fig. 15 represent serialized vertex records or edge records.

13 We do not support multivalued properties directly as intended by TinkerPop. However, we do support regular properties of List or Set types.

14 The Graph Computer is the entry point to the distributed Online Analytics Processing (OLAP) API. Support for this feature may be added in future versions of ChronoGraph.

15 Transactions are an optional feature in TinkerPop 3.

16 Vertices that have been deleted by transaction *t1* while being modified concurrently by transaction *t2* do not disappear from the graph; they remain as *Ghost*s.

17 Half Edges refer to the situation where an edge is only traversable and visible in one direction, i.e., the *out*-vertex lists the edge as outgoing, but the *in*-vertex does not list it as incoming, or vice versa.

18 https://www.osgi.org/.

19 https://mvnrepository.com/artifact/com.github.martinhaeusler/org.chronos.chronosphere.

20 https://docs.jboss.org/hibernate/orm/3.3/reference/en/html/queryhql.html.

21 In Ecore, the operation eGet(...) can be used to access references and attributes. While EReferences always point to other EObjects, EAttributes can have arbitrary values. Thus, eGet(...) in EQuery returns an untyped stream of objects.

22 https://flywaydb.org/.

23 http://www.liquibase.org/.

24 We use the term “client” to refer to application code that operates on top of ChronoSphere. This can be a remote method invocation, another thread or simply a method call to the public API.

25 www.txture.io.

26 https://groovy-lang.org.

27 https://git.io/vxZl3.

28 https://git.io/vxZWp.

29 https://docs.jboss.org/hibernate/orm/3.3/reference/en/html/queryhql.html.

30 https://wiki.eclipse.org/Acceleo/Acceleo_Operations_Reference.

31 https://www.iteraplan.de/en.

32 https://wiki.eclipse.org/CDO.

33 https://www.mongodb.com/.

34 https://www.nomagic.com/products/teamwork-server.

35 https://www.nomagic.com/products/teamwork-cloud.

36 https://osmc.nomagic.com/19.0/swagger/index.html#.

37 https://greycat.ai.

38 https://www.bmc.com/it-solutions/cmdb-configuration-management.html.

39 https://www.servicenow.com/.

40 http://cmshelpcenter.saas.hp.com/CMS/10.30/ucmdb-docs/docs/eng/doc_lib/Content/DIC_Guide.htm.
